# Cryo-EM structures of human RNA polymerase I

**DOI:** 10.1038/s41594-021-00693-4

**Published:** 2021-12-09

**Authors:** Agata D. Misiaszek, Mathias Girbig, Helga Grötsch, Florence Baudin, Brice Murciano, Aleix Lafita, Christoph W. Müller

**Affiliations:** 1 Structural and Computational Biology Unit, European Molecular Biology Laboratory (EMBL), Heidelberg, Germany; 2 Candidate for joint PhD degree from EMBL and Heidelberg University, Faculty of Biosciences, Heidelberg, Germany; 3 European Molecular Biology Laboratory, European Bioinformatics Institute (EMBL–EBI), Cambridge, UK

**Keywords:** Electron microscopy, Transcription, Cancer, Cryoelectron microscopy, Structural biology

## Abstract

RNA polymerase I (Pol I) specifically synthesizes ribosomal RNA. Pol I upregulation is linked to cancer, while mutations in the Pol I machinery lead to developmental disorders. Here we report the cryo-EM structure of elongating human Pol I at 2.7 Å resolution. In the exit tunnel, we observe a double-stranded RNA helix that may support Pol I processivity. Our structure confirms that human Pol I consists of 13 subunits with only one subunit forming the Pol I stalk. Additionally, the structure of human Pol I in complex with the initiation factor RRN3 at 3.1 Å resolution reveals stalk flipping upon RRN3 binding. We also observe an inactivated state of human Pol I bound to an open DNA scaffold at 3.3 Å resolution. Lastly, the high-resolution structure of human Pol I allows mapping of disease-related mutations that can aid understanding of disease etiology.

## Abstract (web summary)

Here the authors structurally investigate elongating human RNA polymerase I at 2.7 Å using cryo-electron microscopy, as well as an RNA polymerase I open complex at 3.3 Å and bound to initiation factor RRN3 at 3.2 Å.

## Main

RNA polymerase I (Pol I) is one of three eukaryotic RNA polymerases and is specialized in the transcription of ribosomal RNA (rRNA)^1^. rRNA constitutes 80–90% of the total RNA mass in mammalian cells^2^. Owing to the high-energy expense imposed by rRNA transcription, Pol I activity needs to be tightly regulated. Pol I transcription is the first step in ribosome biogenesis and thus plays a key role in cellular homeostasis^3^. Upregulation of Pol I activity is required for cancer cells to proliferate. Therefore, Pol I transcription is a promising drug target for a range of cancer types^4^. Several drugs acting on Pol I cofactors are already in clinical use, while other small molecules directly targeting Pol I are in clinical trials^3^. Transcriptional activity at the rDNA loci may compromise gene integrity, which promotes ageing^5^. Concomitantly, partial inhibition of Pol I has been associated with increasing longevity^6^. High rates of rRNA transcription are also observed in pluripotent stem cells^5^ and any misregulation in the function of the Pol I machinery during development can lead to diseases resulting from impaired ribosome biogenesis and function, collectively named ribosomopathies^7^. Mutations causing acrofacial dysostosis (AD) and some mutations causing Treacher Collins syndrome (TCS) have been mapped to the Pol I core^8,9^. Other mutations causing TCS, as well as many mutations associated with hypomyelinating leukodystrophy (HLD), are also found in the subunits shared between Pol I and Pol III, making it difficult to distinguish whether functional impairment of Pol I or Pol III is causing the diseases^10–12^. A detailed understanding of Pol I structure and function in humans is therefore required to better understand its role in development, aging, and the etiology of diseases.

Pol I shares its general architecture with other eukaryotic DNA-dependent RNA polymerases^13^, which comprise of a homologous core bound by the stalk subcomplex^13^. In addition, Pol I and Pol III have stably integrated subunits homologous to general transcription factors of Pol II, namely TFIIE and TFIIF^14^. Pol I is the second largest eukaryotic RNA polymerase. While yeast Pol I comprises 14 subunits^14,15^, in humans only homologs of 13 subunits have been identified^16^. For transcription to take place, Pol I needs to bind to the promoter sequence in the context of a preinitiation complex (PIC). Pol I bound by RRN3 associates with the five-subunit selectivity factor 1 (SL1) containing the TATA-box binding protein (TBP), which is activated by the upstream binding factor (UBF) to allow specific rRNA transcription initiation^16^. All components of the human Pol I PIC are regulated by post-translational modifications to regulate the rate of rRNA production in response to the cell cycle, growth factors, nutrient availability, and stress^17–22^.

While we have a detailed understanding of the structure and transcription cycle of yeast Pol I^14,15,23–32^, structural insights into human Pol I have been lacking. Here we report cryo-electron microscopy (cryo-EM) structures of human Pol I bound to different nucleic acid scaffolds as well as in complex with initiation factor RRN3 at 2.7 to 3.3 Å resolution. Obtained insights expand our understanding of human Pol I function and lay the ground for further studies of its regulation and role in disease.

## Results

### Structure of elongating human Pol I

**Fig. 1:**
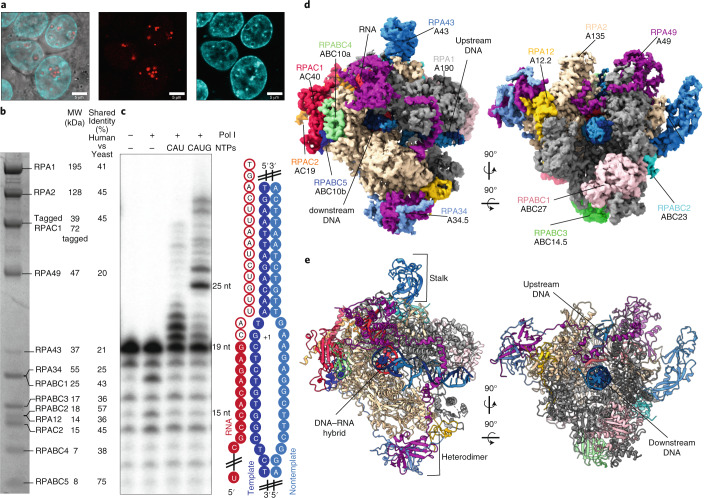
Structure of human Pol I. **a**, Confocal images of the HEK293T cell line with endogenously tagged subunit RPAC1, showing localization to the nucleolus. Overlay of the transmission image with the mCherry signal (red, middle) and live Hoechst dye staining the DNA (cyan, right). Scale bars, 5 μm; *n* = 1. **b**, Coomassie-stained SDS–PAGE of the purified human Pol I; representative gel of 15 independent purifications. Identities of the labeled bands were confirmed by mass spectrometry (for details see Supplementary Table 1). The percentage sequence identity shared between yeast and human is shown on the right. **c**, In vitro primer extension assay confirming the activity of the purified Pol I. 5′ radioactively tagged 19-base RNA primer (red) was assembled with the DNA scaffold (blue) with an artificial open bubble introduced by a mismatch (cartoon representation). Purified Pol I was incubated with the nucleic acid scaffold in the presence of NTPs, as indicated at the top of the gel. For experimental details, see Methods. The experiment was replicated three times. **d**, Cryo-EM map of the Pol I EC (composite Map C in Table 1), colored according to its subunit composition. Subunits are labeled with the human nomenclature and (below) with the yeast counterpart. **e**, Structural model of the Pol I EC with the functional subdomains (stalk and heterodimer) indicated. Source data

**Extended Data Fig. 1:**
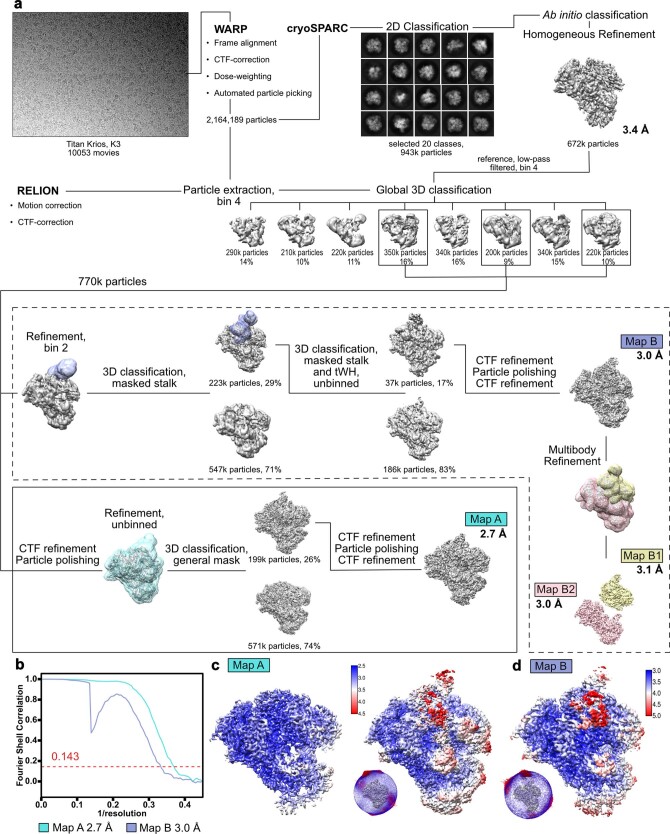
Pol I EC data processing strategy. **a**, Processing pipeline for the Pol I EC dataset. Details are described within the STAR Method Details section. Transparent surfaces show masks used for the 3D classification (violet and cyan) or multi-body refinement (yellow and pink)^84^. Shown are unsharpened cryo-EM maps with threshold levels individually adjusted to show relevant features. Reported resolutions were obtained via RELION post-processing. Annotated maps correspond to the maps reported in Table 1 and used for model building. **b**, FSC curve for the Pol I EC showing the final average resolution for the map A (2.7 Å) and B (3.0 Å) (FSC = 0.143). **c**,**d**, Local resolution estimation of the Pol I EC for **c** Map A and **d** Map B obtained with the script implemented in RELION. Angular distribution plot of all particles contributing to the structures is shown next to the local resolution maps. **c**, The map at a higher threshold (left) shows the high resolution core, while at lower threshold values (right), lower resolution peripheral subunits become visible.

For cryo-EM structure determination, we purified human Pol I from HEK293T suspension cells where subunit RPAC1 was endogenously tagged by CRISPR–Cas9 (ref. ^33^). The introduced tag carries the mCherry fluorescent protein, allowing us to confirm the correct localization of Pol I to the nucleoli in the engineered cell line (Fig. 1a). The quality of purified Pol I was assessed by SDS–PAGE, and the identity of all subunits was confirmed by mass spectrometry (Fig. 1b and Supplementary Table 1). Additionally, we confirmed the transcriptional activity of the obtained human Pol I through an in vitro RNA primer extension assay (Fig. 1c). Subsequently, we used the DNA–RNA scaffold, mimicking the elongation transcription bubble, for the cryo-EM structure determination of the Pol I elongating complex (Pol I EC). The human Pol I EC was resolved at 2.7 Å resolution (Extended Data Fig. 1). To resolve the more flexible, peripheral parts of the complex, we used focused classification and multibody refinement, which yielded three additional partial cryo-EM maps (Extended Data Fig. 1 and Table 1).

**Table 1:** Cryo-EM data collection, refinement, and validation statistics

	Map A (EMDB 12795) EC Pol I (PDB 7OB9)	Map B (EMDB 12795) Stalk-tWH-focused classification	Map B1 (EMDB 12795) Multibody refinement upper clamp	Map B2 (EMDB 12795) Multibody refinement core	Map C (EMDB 12795) Composite
**Data collection and processing**					
Magnification	105,000	105,000	105,000	105,000	105,000
Voltage (kV)	300	300	300	300	300
Electron exposure (e^–^/Å^2^)	50.9	50.9	50.9	50.9	50.9
Defocus range (μm)	1.0–2.5	1.0–2.5	1.0–2.5	1.0–2.5	1.0–2.5
Pixel size (Å)	0.822	0.822	0.822	0.822	0.822
Symmetry imposed	C_1_	C_1_	C_1_	C_1_	C_1_
Initial particle images (no.)	2,171,736	2,171,736	2,171,736	2,171,736	2,171,736
Final particle images (no.)	198,822	37,180	37,180	37,180	−
Map resolution (Å)	2.7	3.0	3.1	3.0	−
FSC threshold	0.143	0.143	0.143	0.143	−
Map resolution range (Å)	2.55–4.50	3.03–5.62	3.13–5.81	3.04–5.10	−
**Refinement**					
Initial model used (PDB code)	4C3I, 7AE1, 6LHR, 5M5X, 6RQT				
Model resolution (Å)	3.30				
FSC threshold	0.5				
Map sharpening *B* factor (Å^2^)	−67				
Model composition					
Non-hydrogen atoms	36,471				
Protein residues	4,412				
Nucleotide residues	69				
Ligands	6× Zn, 1× Mg				
*B* factors (Å^2^)					
Protein	109.26				
Ligand	115.09				
R.m.s. deviations					
Bond lengths (Å)	0.008				
Bond angles (°)	1.26				
Validation					
MolProbity score	1.60				
Clashscore	4.64				
Poor rotamers (%)	0.31				
Ramachandran plot					
Favored (%)	94.70				
Allowed (%)	5.25				
Disallowed (%)	0.05				

	Map D (EMDB 12796) Pol I–RRN3	Map E (EMDB 12796) Pol I–RRN3-focused refinement (PDB 7OBA)	Map F (EMDB 12797) Pol I OC (PDB 7OBB)	Map G (EMDB 12797) Pol I OC nucleic acid focused classification
**Data collection and processing**				
Magnification	130,000	130,000	130,000	130,000
Voltage (kV)	300	300	300	300
Electron exposure (e^–^/Å^2^)	41.26	41.26	41.26	41.26
Defocus range (μm)	0.5–2.25	0.5–2.25	0.5–2.25	0.5–2.25
Pixel size (Å)	0.822	0.822	0.822	0.822
Symmetry imposed	C_1_	C1	C_1_	C_1_
Initial particle images (no.)	2,628,144	2,628,144	2,628,144	2,628,144
Final particle images (no.)	169,513	260.363	175,912	164,436
Map resolution (Å)	3.2	3.1	3.3	3.3
FSC threshold	0.143	0.143	0.143	0.143
Map resolution range (Å)	3.0–7.0	2.9–7.3	3.0–5.9	3.0–6.7
**Refinement**				
Initial model used (PDB code)		4C3I, 7AE1	4C3I, 7AE1, 5M5W, 6RUO	
Model resolution (Å)		3.1	3.6	
FSC threshold		0.5	0.5	
Map sharpening *B* factor (Å^2^)		−103	−91	
**Model composition**				
Non-hydrogen atoms		35,684	33,051	
Protein residues		4,399	4,113	
Nucleotide residues		—	17	
Ligands		7× Zn	7× Zn	
***B*** **factors (Å**^2^**)**				
Protein		65.99	75.19	
Ligand		88.12	119.54	
**R.m.s. deviations**				
Bond lengths (Å)		0.0111	0.0097	
Bond angles (°)		141	1.37	
**Validation**				
MolProbity score		1.77	1.76	
Clashscore		5.33	5.21	
Poor rotamers (%)		0.41	0.50	
**Ramachandran plot**				
Favored (%)		91.96	92.24	
Allowed (%)		7.99	7.74	
Disallowed (%)		0.05	0.02	

We assigned cryo-EM densities to 13 Pol I subunits (Fig. 1d) and built the atomic model of the human Pol I EC (Fig. 1e). The general architecture consists of a horseshoe-shaped core bound by a stalk formed by the RPA43 subunit, resembling yeast Pol I. The core is further complemented by a TFIIE/F-like heterodimer, which consists of RPA34 and RPA49 (ref. ^34^). The carboxy terminus of RPA49 harbors a TFIIE-like tandem-winged-helix (tWH) domain^13,34,35^ observed close to the RNA exit tunnel (Fig. 1d,e). The well-resolved DNA–RNA scaffold enabled us to build a large portion of the downstream double-stranded DNA, the DNA–RNA hybrid, and the RNA in the exit tunnel, as well as a portion of the upstream DNA (Fig. 1d,e). Overall, the general architecture of Pol I is conserved between yeast and humans, despite the low sequence identity ranging from 20–45% for Pol I-specific subunits (Fig. 1b).

### Double-stranded RNA in the exit tunnel

**Fig. 2:**
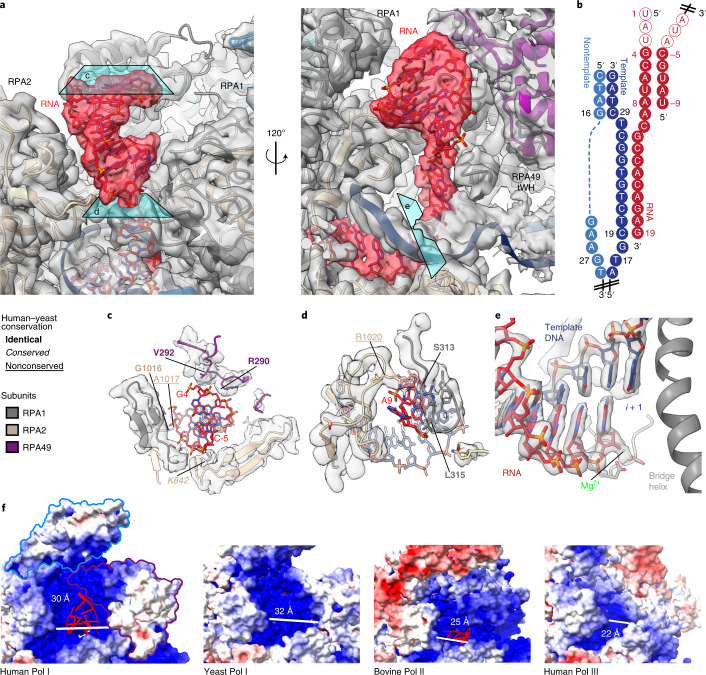
RNA exit tunnel is able to accommodate a double-stranded RNA helix. **a**, Close-up view of the RNA exit tunnel. Cryo-EM density from Map B1 for the RNA (red) and the surrounding (gray) is shown. The tWH domain of RPA49 in the left panel and a section of subunit RPA2 obscuring the RNA exit tunnel in the right panel are hidden. **b**, Schematic representation of the nucleic acid scaffold. A second RNA primer (right) base pairs to the primer annealed to the DNA scaffold, forming a double-stranded helix owing to the sequence complementarity. The empty circles correspond to the RNA bases not visible in the cryo-EM density. **c**,**d**, Cross-sections through the RNA exit tunnel as indicated in **a**. Cryo-EM density corresponding to the protein components forming the tunnel is shown in gray. Residues within 5 Å of the RNA are shown represented as sticks. Residues making contacts with the RNA are annotated. Conservation between yeast and human Pol I residues is indicated as in the legend on the left of **c**. **e**, Cryo-EM density corresponding to the DNA–RNA hybrid is shown in gray. The bridge helix is in cartoon representation, and active site Asp residues are represented as sticks. **f**, Electrostatic charge distribution on the surface of (left to right) human Pol I EC, yeast Pol I (PDB: 5M64 (ref. ^25^)), bovine Pol II (PDB: 5FLM^38^), and human Pol III (PDB: 7AE1 (ref. ^33^)). RNA in the exit tunnel is shown as a cartoon (red). In the left panel, outlines of the RPA43 subunit (blue) and tWH of the RPA49 (purple) are shown. The tunnel width was measured using ChimeraX from backbone to backbone using human Pol I residues RPA1-S508 to RPA49-K362, bovine Pol II residues RPB1-K434 to RPB2-Q838, human Pol III residues RPC1-Y434 to RPC2-A798, and yeast Pol I residues A190-G548 to A49-N354.

**Extended Data Fig. 2:**
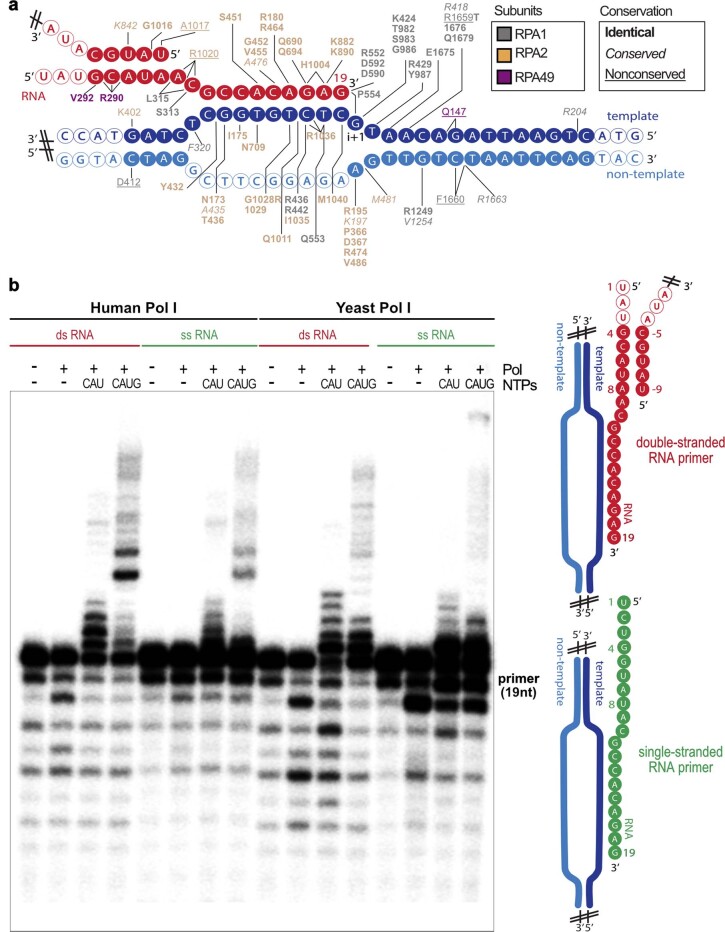
Pol I interactions with nucleic acids. **a**, Residues contacting the nucleic acid scaffold in human Pol I. The nucleic acid scaffold is presented in cartoon representation with colored circles marking nucleotides visible in the cryo-EM density and open circles marking nucleotides lacking cryo-EM density that have not been included into the final model. Human Pol I residues making contacts with nucleotides within 4 Å radius are depicted. Residues are colored according to the subunits they are a part of and their level of conservation between human and yeast Pol I is marked. **b**, Comparison of the transcriptional activity between human and yeast Pol I using an RNA primer that dimerizes and a single-stranded RNA primer. Used nucleic acid scaffolds in the cartoon representation are shown on the right. The double-stranded RNA primer (red) has been used throughout the study and the single stranded primer (green) has been designed to have similar base composition. Both primers were radioactively labeled on the 5′ end and annealed to the DNA scaffold as described in Methods. Presence or absence of Pol I in the assay as well as addition of nucleotides is indicated on top. Source data

The DNA–RNA scaffold mimicking the elongation bubble contains a 19-nucleotide RNA primer and a 43-bp DNA duplex with a mismatch over 12 nucleotides (Extended Data Fig. 2a). In the active site, we confidently assigned positions of all bases in the DNA–RNA hybrid. Further, we traced the RNA backbone extending into the RNA exit tunnel over a length of 16 nucleotides in total. This is remarkably different compared with similar structures of elongating RNA polymerases. In the structures of human Pol III, only 5 (ref. ^36^) to 6 (ref. ^33^) nucleotides were visible, while in yeast Pol I, between 7 and 13 nucleotides were traced from the active site extending into the RNA exit tunnel^25^. To our surprise, the cryo-EM density map showed features similar to those of double-stranded RNA in the exit tunnel (Fig. 2a). The 5′ end of the RNA oligonucleotide used is self-complementary to the 3′ end of the exiting RNA oligonucleotide (Fig. 2b), and thus we placed an ideal double-stranded RNA helix (A-form) formed by base-pairing of two separate RNA oligonucleotides into the exit tunnel that fitted well into the cryo-EM density map (Fig. 2a). So far, RNA secondary structure elements in the exit tunnel of RNA polymerases have only been captured in bacterial RNA polymerase in a paused state^37^. Human Pol I EC structure represents a post-translocated state with an empty *i* + 1 site, allowing accommodation of the incoming nucleotide (Fig. 2e). Structural features in the RNA exit tunnel as well as majority of the Pol I residues contacting the RNA are conserved between yeast and human (Fig. 2c,d and Extended Data Fig. 2a). After the nascent RNA strand separates from the DNA template strand, it is directed into a narrow tunnel (Fig. 2d), measuring ~16 Å in diameter. The backbone of the emerging RNA strand is directed by the contacting residues R1020 and L315 of RPA2 and RPA1, respectively (Fig. 2d). Subsequently, the RNA exit tunnel widens up to ~30 Å in diameter, conducive to the accommodation of the double-stranded RNA (Fig. 2c,f). The funnel formed by RPA1, RPA2, and the tWH domain of RPA49 is highly positively charged (Fig. 2f)^37^. Indeed, the tunnel in Pol I is wider, and the positively charged patch is larger, than are those of mammalian Pol II^38^ and Pol III^33^ (Fig. 2f). Also unique to Pol I is the position of the RPA49 tWH domain, which further extends the RNA exit tunnel with its positively charged surface (Fig. 2f).

The specialized adaptations of Pol I in the RNA exit tunnel may facilitate nascent RNA folding and thus positively influence the transcription rate^39^ (Extended Data Fig. 2b). Stable RNA structures correlate with a high transcription rate because they prevent the transcript’s re-entry into the active site, required for backtracking. The positive effect of RNA folding on elongation rates seems to be conserved among all RNA polymerases^39^. Additionally, our results show that the human Pol I might stabilize the RNA structures in the exit tunnel more efficiently than can human Pol III, where no RNA structure could be observed despite the use of the same RNA primer^33^. High transcription rates might be especially important for the transcription of the rDNA repeat owing to its length (13 kb in humans and 6.9 kb in *Saccharomyces* *cerevisiae*)^1^. rRNA folds co-transcriptionally and undergoes complex processing along the ribosome biogenesis pathway^40^, requiring specific RNA structures. The wide and positively charged RNA exit tunnel observed in Pol I allows the formation of such RNA structures.

### Human Pol I stalk contains a single subunit

**Fig. 3:**
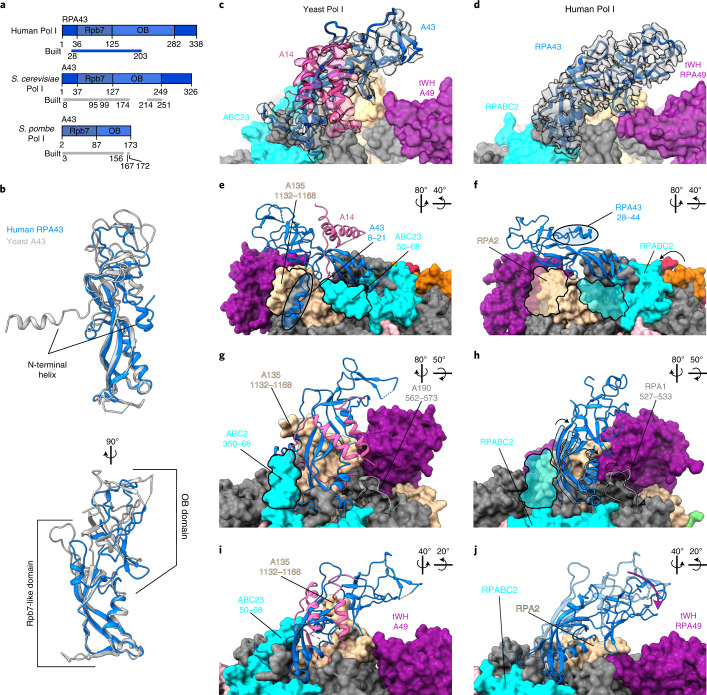
Human Pol I stalk is made out of only one subunit. **a**, Domain composition of the human RPA43 (top) and its yeast A43 homologs: *S. cerevisiae* (middle; PDB 5M64)^25^ and *S. pombe* (bottom; PDB 7AOE)^43^. The colored bar indicates the region modeled in the structures denoted as ‘built’. Rpb7, Rpb7-like domain; OB, oligonucleotide/oligosaccharide binding domain. **b**, Structure overlay of the human RPA43 subunit with its yeast (*S. cerevisiae*) homolog (PDB: 5M64)^25^. **c**–**j**, Yeast Pol I stalk (PDB: 5M64)^25^ (**c**,**e**,**g**,**i**) compared with human Pol I stalk (**d**,**f**,**h**,**j**). Stalk subunits A43 (yeast) and RPA43 (human) (blue) and yeast A14 (pink) are shown in the cartoon representation, while the rest of the Pol I is shown in the surface representation, colored as in Fig. 1. The rotations of the views between the panels are indicated in the top right corner of the panels. **c**,**d**, Cryo-EM density corresponding to A43/RPA43 (gray) and A14 (pink) is shown in a transparent representation. **e**,**f**, Structurally homologous N-terminal portions of the A43 and RPA43 subunits are circled. **g**,**h**, The yeast A190 loop (562–573) structure (**g**) is overlaid on the human structure (**h**) as a transparent cartoon. **h**, In humans, the RPA1 loop (527–533) (cartoon representation, solid) is less structured and folds away from the stalk (bottom black arrow). The lower part of the stalk in humans tilts in the same direction (black arrows) when compared with yeast (overlaid transparent cartoon of the lower part of the yeast stalk). **e**–**h**, Outlines of the yeast specific extensions in A135 and ACB23 marked in **e** and **g** are overlaid onto the human structure in **f** and **h**, respectively. **i**,**j**, The human Pol I stalk leans (purple arrow) toward the RPA49 tWH domain when compared with the yeast structure, which is overlaid as a transparent cartoon.

The stalk of yeast Pol I consists of two subunits: A43, homologous to Rpb7 in Pol II and C25 in Pol III, and A14, homologous to Rpb4 in Pol II and C17 in Pol III^14,41,42^. The A43 stalk subunit has a similar domain architecture in yeast and humans, with the core anchoring Rbp7-like domain being especially conserved (Fig. 3a,b). A larger portion of the C-terminal moiety of subunit A43 could be assigned in *S. cerevisiae* and *Schizosaccharomyces* *pombe* compared with human RPA43, likely owing to higher flexibility of the OB domain in humans (Fig. 3a,b). Nonetheless, sequence homology searches did not identify any homologous proteins to the yeast subunit A14 in humans. The cryo-EM structure of human Pol I reveals that the stalk solely contains RPA43 (Fig. 3d), whereas in the stalk of yeast Pol I, clear cryo-EM density corresponding to two helices of the subunit A14 is visible (Fig. 3c). Instead, in the human RPA43 subunit, the amino-terminal portion (residues 28–44) forms a helix which points away from the core, occupying the same space as the yeast A14 in the stalk (Fig. 3e,f). The corresponding region in yeast A43 instead points in the opposite direction and is bound to the Pol I core by a 36-residue extension of subunit A135, which is absent in the corresponding human subunit RPA1 (Fig. 3e,f). Consequently, in human Pol I, the binding interface between the stalk and core is smaller than in yeast Pol I. In addition, the lack of the structured helical extension in the N-terminal portion of subunit RPABC2 and the presence of a more flexible loop (residues 527–533) in subunit RPA1 (Fig. 3g,h) permit tilting of the stalk towards the tWH domain of RPA49 (Fig. 3i,j).

**Extended Data Fig. 3:**
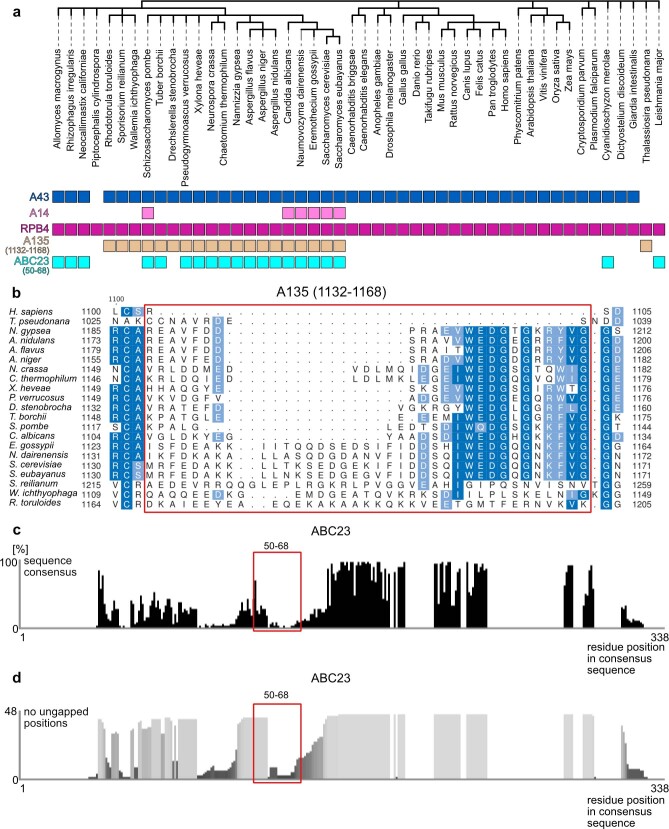
Phylogenomic analysis of the stalk subunits and extensions from A135 and ABC23 contacting the stalk. **a**, Analysis of A14 and A43 Pol I stalk subunits, smaller stalk subunit RPB4 from Pol II and extensions from subunits A135 (1132–1168) and ABC23 (50–68) as indicated in Fig. 2. Filled square indicates presence of a homologous protein in the corresponding species. Color code as in Fig. 3. Genes are annotated per *S. cerevisiae* nomenclature. For UniProt identifiers of the identified homologues see Supplementary Table 2. **b**, Sequence alignment of subunit A135 across species where the *S. cerevisiae* 1132–1168 insert was identified. Human sequence of RPA2 subunit is included in top row for comparison. Sequences are sorted by alignment score. **c**, Sequence consensus score plotted across the length of the consensus sequence for the ABC23 subunit. Percentage of sequences with the consensus residue is shown. **d**, Occupancy plot for the ABC23 subunit showing the number of ungapped residues in each position of the consensus alignment. Total of 48 sequences were aligned. **b**–**d**, The red box marks the location of the inserts of interest. Numbering in reference to *S. cerevisiae* sequence.

The absence of an A14 homolog in humans prompted us to conduct an extended phylogenetic analysis (Extended Data Fig. 3 and Supplementary Table 2). We found that, although the presence of the second stalk subunit is conserved throughout the eukaryotic tree of life for Pol II, in the case of Pol I, it is found only in some species from a division of fungi, ascomycota (Extended Data Fig. 3a). Consequently, previous studies using *S. cerevisiae*^14,15^ or *S. pombe*^43^ have focused on outliers within the tree of life. We next investigated the phylogenomic conservation of the yeast extensions of subunits A135 (residues 1135–1168) and ABC23 (residues 50–68), which contact the stalk (Fig. 3g). Since the subunits A135 and ABC23 are highly conserved, we created sequence alignments of the full-length proteins (Supplementary Data 1) and checked for the presence of insertions (Extended Data Fig. 3a). We found that the insertion in the A135 (1135–1168) appeared within fungi (Extended Data Fig. 3a,b), which correlates with the presence of the two-subunit stalk in those species. The N terminus of the ABC23 subunit is less conserved (Extended Data Fig. 3c,d), and thus its relation to the stalk subunits requires further investigation. The absence of an A14 homolog in human Pol I likely renders the stalk more flexible, which is consistent with our cryo-EM data and could play a role in the association of transcription factors, such as RRN3, via conformational selection.

### Structured extensions bind the heterodimer to the core

**Extended Data Fig. 4:**
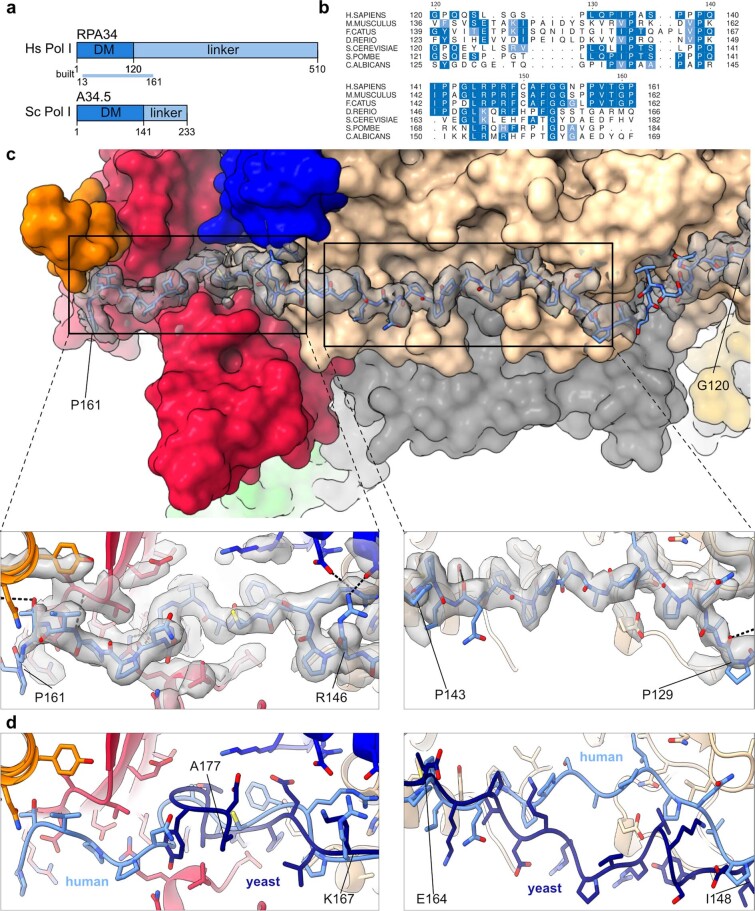
The RPA34 extension tethers the heterodimer to the Pol I core. **a**, Cartoon domain representation of the domain composition of the human RPA34 (top) and its yeast homologue A34.5 (bottom). The colored bar indicates the region modeled in the structure denoted as ‘built’. DM – dimerization domain. **b**, Multiple sequence alignment of the modeled portion of the RPA34 extension between different species listed on the left. Dark blue marks identical residues and light blue similar residues. Dots mark gaps in the sequence. **c**, RPA34 extension path (stick representation) on the Pol I surface (surface representation). Cryo-EM density, corresponding to the extension is shown in gray, transparent representation. Lower panels show the close up view of the extension (stick representation) surrounded by the Pol I core (cartoon representation). Residues from the Pol I core within the 5 Å radius from the linker are shown as sticks. Putative hydrogen bonds are shown as black dotted lines. **d**, Superposition of the yeast A34.5 extension from PDB: 4C3I2^19^ (navy) onto the human structure shown in the panel above.

The human Pol I heterodimer consists of subunits RPA34 and RPA49 and is structurally and functionally related to TFIIF/TFIIE in the Pol II system^26,27,44^. The overall architecture of the heterodimer, consisting of a dimerization module and long extensions that bind the core, is conserved across species^14,34,35^. Yet the sequence identity of this subcomplex between yeast and human is only 20% and 25% for RPA49 and RPA34, respectively (Fig. 1b). Subunit RPA34 in humans is over twice as large as in yeast (Extended Data Fig. 4a), owing to a disordered C-terminal tail. Its first ~40 amino acids (residues 120–161 in humans) can be assigned to a density running along the core of Pol I, and docking into a cleft within subunit RPAC1 (Extended Data Fig. 4c). While the general path of the extension is conserved (Extended Data Fig. 4c,d), its sequence is highly divergent (Extended Data Fig. 4b,c)^14,15^. In human RPA34, the extension is proline-rich and adopts a rigid conformation comprising several kinks contacted by core residues (Extended Data Fig. 4c, close-up panels). Restricted conformational flexibility of proline-rich elements on one side creates a continuous hydrophobic stretch, while the opposite side harbors many sites that are accessible for hydrogen bonding, which together serve as a favorable platform for protein-protein interactions^45^. Instead, in yeast A34.5, the extension contains more charged residues (Extended Data Fig. 4b,d), which contact core residues and form hydrogen bonds. Taken together, the different sequences accomplish the same function of tightly anchoring the heterodimer to the Pol I core.

Other possibly diverse roles of the disordered C terminus of RPA34 are not fully understood. While yeast A34 harbors a nucleolar localization signal^46^, human RPA34 has been shown to diffuse out of the nucleolus upon starvation^47^, which might be modulated by post-translational modifications within the C-terminal extension^48^. The C-terminal extension also appears to affect the rate of rRNA transcription and was suggested to bind SL1 on the basis of in vivo studies^47^. RPA34 was additionally proposed to interact with UBF, possibly through multiple binding sites throughout the C-terminal extension^49^.

**Extended Data Fig. 5:**
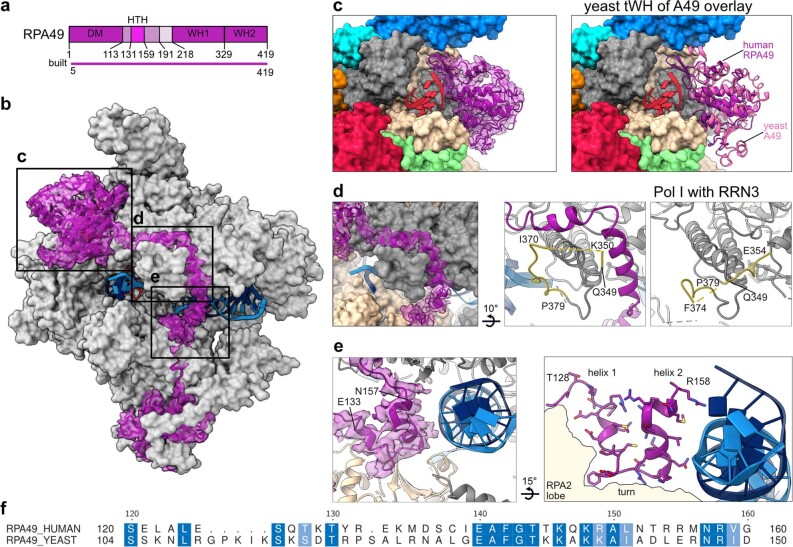
RPA49 linker runs along the Pol I clamp to position the tWH domain. **a**, Cartoon domain representation of the RPA49 subunit. The colored bar indicates the region modeled in the structure denoted as ‘built’. DM – dimerization domain; HTH – helix-turn-helix motif; WH – winged helix domain. **b**, Overall arrangement of the RPA49 subunit (purple, cartoon representation fitted into the cryo-EM density extracted from Map C) on the surface of Pol I (grey, surface representation). The DNA scaffold is shown as a cartoon. **c**, Positioning of the tWH domain (purple, cartoon representation fitted into the corresponding cryo-EM density) near the RNA exit tunnel. The Pol I core is shown in a surface representation colored by subunit. Right panel: yeast tWH domain (pink, cartoon representation) from PDB: 5M64^30^ overlaid onto the human Pol I structure. **d**, Left panel: the linker (purple) wraps around a knob formed by clamp coiled-coils (grey, surface representation). Middle panel: partially disordered loop (gold highlight) connecting the coiled-coils (grey cartoon representation) allows binding of the RPA49 linker (purple cartoon). Right panel: in the structure of human Pol I bound to RRN3, where the RPA49 linker is disordered, the same loop (golden highlight) adopts a conformation that would clash with the RPA49 linker. **e**, HTH motif within the linker (purple with the corresponding cryo-EM density shown in transparent representation) is positioned close to the downstream DNA helix (blue, cartoon representation). Right panel: side chains of the HTH are shown in stick representation (purple). Helix 1 and turn contact the RPA2 lobe (wheat outline), while helix 2 can contact the downstream DNA. **f**, Sequence alignment of the part of RPA49 linker containing the HTH motif between human and yeast. Dark blue marks identical residues and light blue similar residues. Dots mark gaps in the sequence.

Subunit RPA49 is a hybrid between TFIIF and TFIIE, owing to its N-terminal dimerization and C-terminal tWH domain, respectively (Extended Data Fig. 5a)^34,35^. The dimerization domain is anchored to the RPA2 subunit, and the C-terminal tWH domain binds the clamp close to the RNA exit tunnel (Extended Data Fig. 5b). The overall fold and position of the human RPA49 tWH domain is similar to the one found in yeast A49 (Extended Data Fig. 5c). The tWH domain has DNA-binding capability and may change position to contact upstream DNA, as seen in yeast Pol I PIC^27^. Following the positioning of the tWH domain, the linker between dimerization domain and tWH domain runs along the upper clamp and is anchored to a knob formed by the two coiled-coil helices in the RPA1 clamp core (Extended Data Fig. 5d, left). The partially disordered loop (residues 345–383) connecting these two helices changes its conformation upon RPA49 linker binding (Extended Data Fig. 5d). The RPA49 linker further crosses the DNA-binding cleft in close proximity to the downstream DNA (Extended Data Fig. 5e, left). The RPA49 linker harbors a helix-turn-helix (HTH) motif (Extended Data Fig. 5e, right) which is structurally and functionally conserved between yeast and humans, despite its only partial sequence conservation (Extended Data Fig. 5f). It is predicted to bind DNA and was shown to be required for cell proliferation^50^. While mutational studies in vitro show that both helix 1 and helix 2 have DNA-binding capability^50^, in our structure, only helix 2 is poised for interactions with the DNA, whereas helix 1 is bound to the RPA2 lobe (Extended Data Fig. 5e, right).

### Human Pol I bound to RRN3 and in open complex conformation

**Fig. 4:**
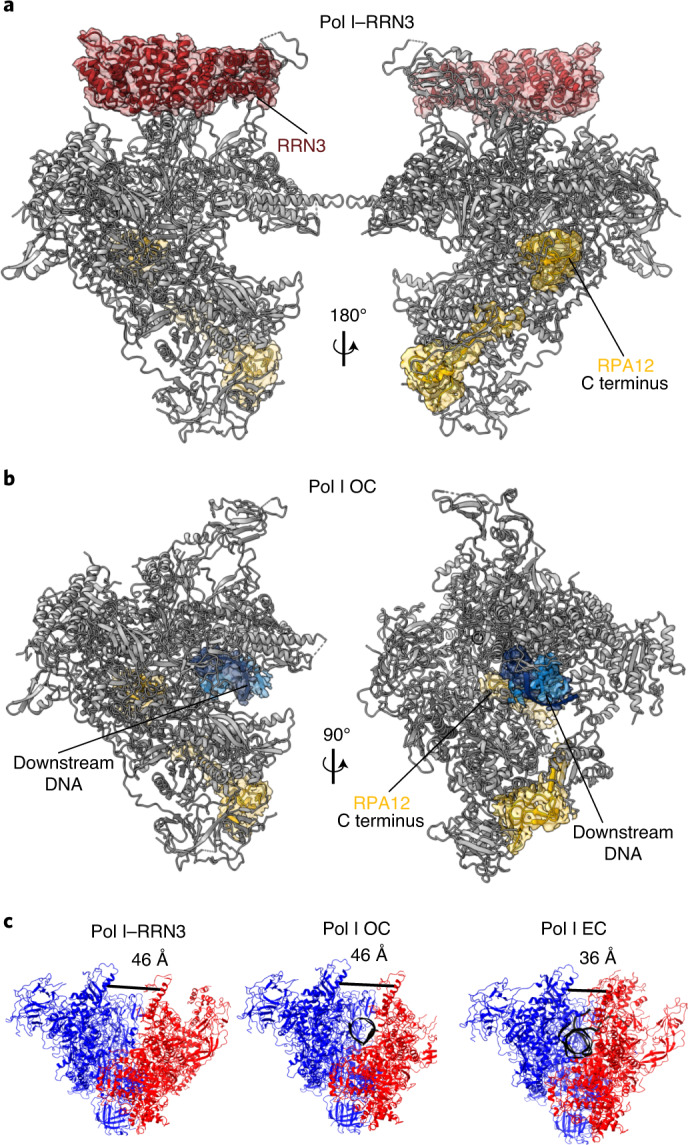
Structure of Pol I–RRN3 and Pol I OC. **a**,**b**, Pol I–RRN3 (**a**) and Pol I OC (**b**). Cryo-EM density for RPA12 and DNA (in the Pol I OC) or newly built subunits (RRN3 in the Pol I–RRN3) is shown as a transparent red, yellow and blue surfaces. **c**, Differences in the cleft width between the three human Pol I structures. The two modules which move with respect to each other are colored in red (upper clamp and stalk) and blue (core and heterodimer). Nucleic acids are colored in black. The distance was measured from backbone to backbone of RPA1-L341 (clamp core) to RPA2-I396 (protrusion) and is shown with a black line.

**Extended Data Fig. 6:**
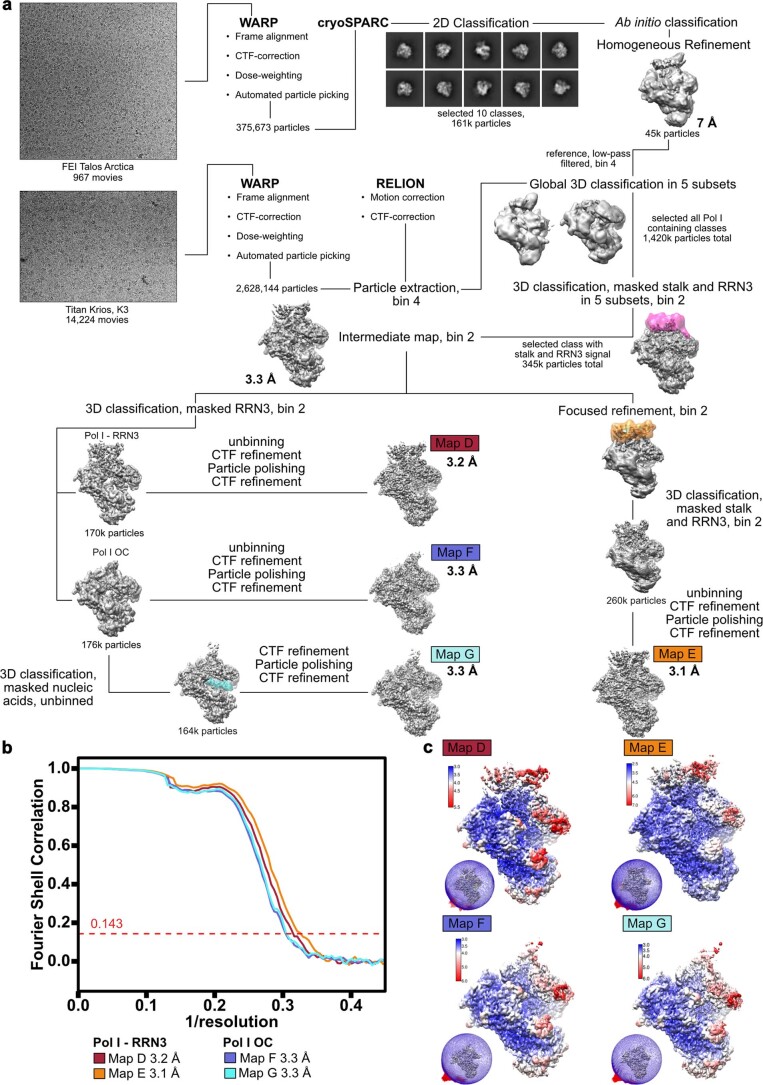
Pol I-RRN3 and Pol I OC dataset processing strategy. **a**, Details of the pipeline are described within the STAR Method Details section. A small initial dataset was collected using FEI Talos Arctica and processed in cryoSPARC to obtain a reference that was used for global 3D classification for the high-resolution dataset collected on Titan Krios. First global 3D classification was performed with the dataset split into 5 subsets and representative selected classes are shown. Transparent surfaces show masks used for the 3D classification (pink and cyan) or for focused refinement (orange). Shown are unsharpened cryo-EM maps with threshold level individually adjusted to show relevant features. Reported resolutions were obtained via RELION post-processing. Annotated maps correspond to the maps reported in Table 1 and used for model building. **b**, FSC curves for the Pol I-RRN3 and Pol I OC maps showing the final average resolution (FSC = 0.143). **c**, Local resolution estimation of Pol I-RRN3 and Pol I OC maps obtained with the RELION implementation. Maps are shown at lower threshold values, so that the lower resolution peripheral subunits become visible. Angular distribution plot of all particles contributing to the structures is shown to the left of each local resolution map.

To further investigate the structure and function of human Pol I, we obtained the structure of Pol I bound to the initiation factor RRN3 (ref. ^51^). Human RRN3 interacts with SL1 and primes Pol I for transcription initiation^52^. We incubated purified Pol I with recombinant human RRN3 in equimolar ratios in the presence of the DNA scaffold devoid of RNA. After extensive classification of the acquired dataset (Extended Data Fig. 6), we obtained two cryo-EM maps corresponding to Pol I bound to RRN3 (Pol I–RRN3) and Pol I bound to the open DNA template, termed open complex (Pol I OC) (Fig. 4a,b). In the Pol I–RRN3 complex, clear cryo-EM density corresponding to RRN3 is visible close to the stalk (Fig. 4a). Owing to the flexibility of the upper clamp, focused refinement was used to improve the map in this region (Extended Data Fig. 6 and Table 1). Focused refinement also alleviated effects of directional resolution anisotropy, improving sphericity from 0.796 to 0.836, as assessed by the 3DFSC program^53^. Despite the lower local resolution, this allowed us to place a human RRN3 homology model into the density with a good fit for the, mostly helical, secondary-structure elements.

The Pol I OC map has a slightly lower resolution (Table 1); notwithstanding, we could confidently build the downstream portion of the DNA scaffold (Fig. 4b). In both structures, the RPA49 tWH domain is disordered (Fig. 4a,b), supporting the notion that it plays a role in clamp closing during transcription elongation^27,28,54^. Insertion of the C-terminal domain of the RPA12 subunit into the active site, observed in the Pol I–RRN3 and Pol I OC structures (Fig. 4a, b), might also be associated with the open clamp conformation. Closing of the clamp by up to 10 Å is associated with tight binding of the nucleic acids and elongation state, while in the transcriptionally inactive states, the clamp is more widely open and flexible (Fig. 4c). In yeast, similar closing of the clamp has been observed, though the clamp closes by 7 Å between Pol I OC and EC states^25^ and by 6 Å between apo Pol I-dimer and Pol I OC states^14,25^. So far, the high clamp flexibility hindered determination of the structure of human apo Pol I, and the dimerization of human Pol I has not been observed. It therefore remains unknown whether the Pol I clamp can open even further.

### Inactive state of Pol I

**Extended Data Fig. 7:**
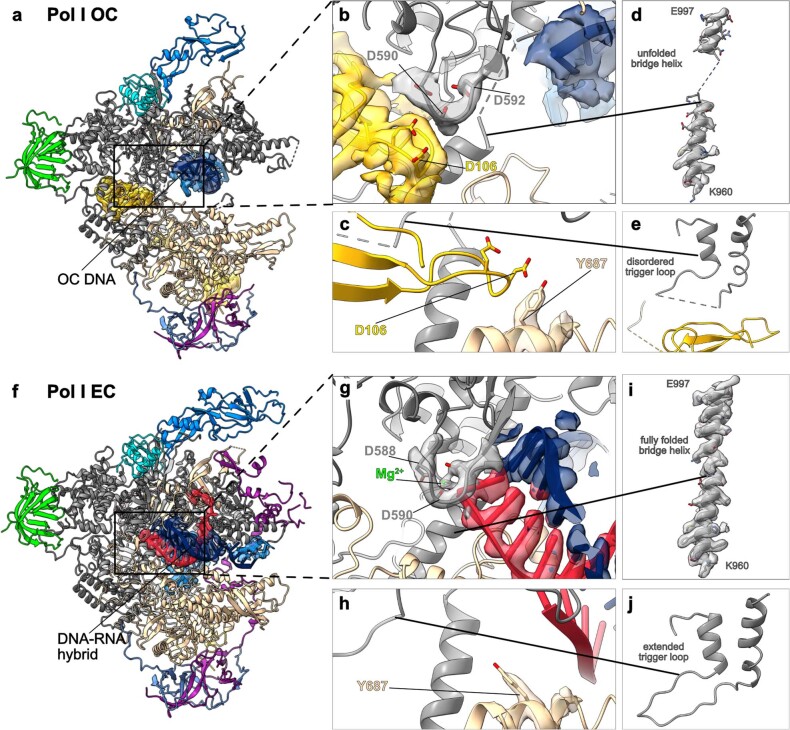
Pol I OC represents an inactive state of Pol I. **a**,**f**, Overview of the Pol I OC (**a**) and Pol I EC structures (**f**) with subunits colored as in Fig. 1. The extracted cryo-EM density for the relevant parts of the structures is shown in transparent representation colored according to the respective subunit. Subunits directly occluding the view of the active site are hidden. **b** Insertion of the RPA12 C-terminal domain (yellow) into the active site. Aspartates in the RPA12 catalytic loop are shown in stick representation. **b**,**g**, Catalytic triad of the aspartates (D588, D590 and D592) is shown in stick representation. **g**, Mg^2+^ ion is marked in green. **c**,**h**, Conformation of the gating tyrosine (Y687) changes between the Pol I OC and Pol I EC. **d**,**e**,**i**,**j**, Functional elements of the active site shown without the occluding elements. They are disordered in Pol I OC (**d** bridge helix and **e** trigger loop), while in the Pol I EC (**i** bridge helix and **j** trigger loop) they are fully folded.

The clamp opening and insertion of the RPA12 C-terminal domain has been associated with the inactive state of Pol I^25^. The C-terminal domain of RPA12 is homologous to TFIIS in the Pol II system and possesses RNA cleavage activity^13,55^. In the apo and OC conformations of yeast Pol I, ordering of the corresponding C-terminal domain of A12 has previously been observed^14,15,25,30^, while it remains disordered in the EC state^25,30^. In the human Pol I OC, the nucleic acid scaffold is bound away from the active site in a nonproductive conformation (Extended Data Fig. 7a,b). We could trace the double-stranded downstream DNA, but the fragmented cryo-EM density lining the DNA-binding cleft did not allow us to confidently build the unwound portion of the DNA, indicating that it is not stably associated with the active site. In the Pol I OC, no density could be unambiguously assigned to the catalytic Mg^2+^, and the density for the catalytic aspartates is also weaker than is that of the Pol I EC (Extended Data Fig. 7b,g). In line with the inactivation during the cleft-expansion mechanism suggested by Engel et al.^15^, one of the catalytic residues, D590 from RPA1, might have flipped out, which could contribute to the poorer cryo-EM density. The functional elements of the active site, such as the bridge helix and the trigger loop are disordered, whereas these elements are fully ordered in the Pol I EC (Extended Data Fig. 7d,e,i,j). The fully extended trigger loop, as seen in the Pol I EC (Extended Data Fig. 7j), would sterically clash with the C-terminal domain of RPA12 (Extended Data Fig. 7e). The insertion of the RPA12 C-terminal domain also triggers a switch of the gating tyrosine (Y687 of RPA2). In the Pol I EC, the gating tyrosine occludes the backtracking funnel, and in Pol I OC it flips forward to avoid the steric clash with residue D106 from RPA12 (Extended Data Fig. 7c,h). Flipping of the gating tyrosine was first observed for yeast Pol II bound to TFIIS. However, in the model proposed by Cheung and Cramer^56^, the gating tyrosine flips to prevent interaction with the backtracked RNA in the reactivation intermediate, thereby enabling TFIIS to fully insert into the funnel. Given that no RNA is present in the Pol I OC sample, we conclude that, at least for human Pol I, the insertion of RPA12, and not the backtracked RNA, induces flipping of the gating tyrosine.

**Extended Data Fig. 8:**
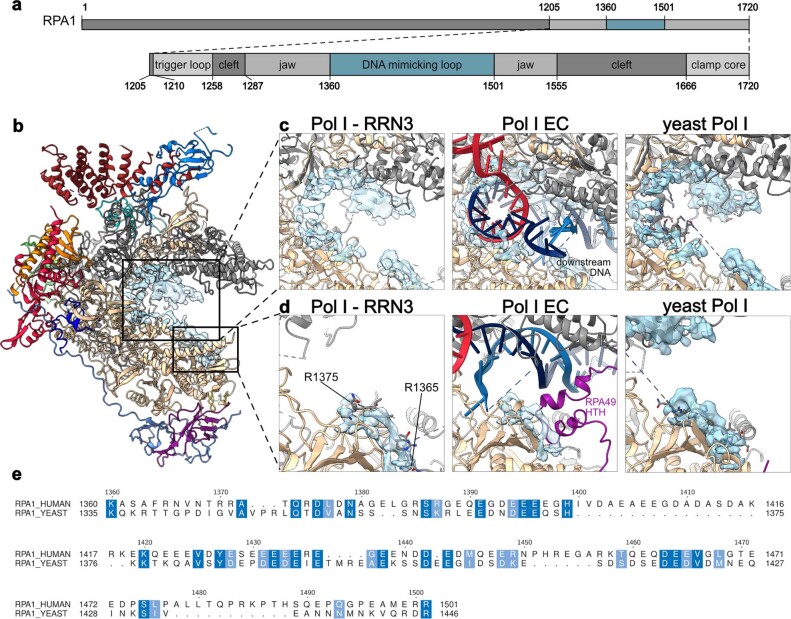
Density corresponding to the DNA mimicking loop in the Pol I-RRN3 map. **a**, Cartoon domain representation of the RPA1 subunit. The C terminus harboring the jaw extension termed ‘DNA-mimicking loop’ is shown on the close-up view. **b**, Overview representation of the Pol I-RRN3 structure. **c**, Close-up view of the additional density in the DNA binding cleft (left panel), which is then overlaid on the Pol I EC structure (middle panel), where it would overlap with the DNA backbone. Density seen in the cleft of human Pol I (left panel) overlaps with the yeast DNA mimicking loop (PDB: 4C3J^19^) (right panel). **d**, Close-up view of the first 10-residues of the DNA mimicking loop (stick representation) which bind to the RPA2 core (left panel). When the cryo-EM density corresponding to those residues is superimposed on the Pol I EC (middle panel) it overlaps with the turn from the HTH motif of subunit RPA49 (purple). The path of the first residues of the DNA mimicking loop is the same in yeast Pol I (right panel). **b**–**d**, Additional density lining the DNA binding cleft in the Pol I-RRN3 structure which likely corresponds to the DNA-mimicking loop is shown as transparent, blue surface. **e**, Sequence alignment of the human and yeast DNA mimicking loop. Dark blue marks identical residues and light blue similar residues. Dots mark gaps in the sequence.

Subunit RPA1 has a large insertion within its jaw (Extended Data Fig. 8a), which is fully disordered in the Pol I EC structure. In the yeast Pol I crystal structures, this insertion harbors the ‘DNA-mimicking loop’ or ‘expander’ that overlaps with the DNA backbone in the DNA-binding cleft^14,15^. In the Pol I–RRN3 conformation, we also observe weak cryo-EM density lining the cleft (Extended Data Fig. 8b). Like in yeast Pol I, when superimposed onto the Pol I EC, this extra density would clash with the DNA backbone (Extended Data Fig. 8c) suggesting that this mode of regulation is conserved between yeast and humans. While there is little sequence identity between yeast and human Pol I in the DNA-mimicking loop region (Extended Data Fig. 8e), in both cases the insertion is negatively charged. Although the density was not of sufficient quality to build an atomic model of the complete human Pol I DNA-mimicking loop, we could unambiguously assign the register for the first 10 residues (1365–1375). When we superimposed its density onto the Pol I EC, it overlapped with the turn from the HTH motif within the RPA49 linker (Extended Data Fig. 8d). Insertion of the DNA-mimicking loop into the cleft could therefore also prevent positioning of the RPA49 linker that assists in closing the clamp in the transition to transcription elongation^25,54^.

### RRN3 binding to Pol I affects the stalk

**Fig. 5:**
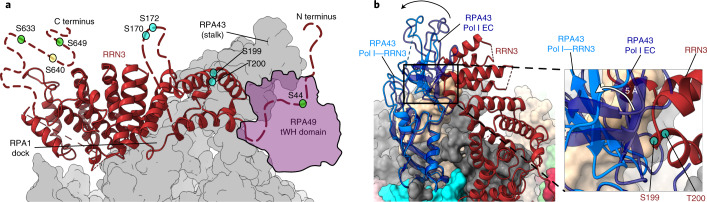
RRN3 binding to human Pol I. **a**, RRN3 (maroon, cartoon representation) binds the Pol I (gray, surface representation) at stalk and RPA1 dock regions. Disordered parts of the human RRN3 that harbor phosphorylation sites are shown with maroon dotted lines (not to scale). Phosphorylation sites are marked with circles: activating phosphorylation sites in green^21,22^, inactivating phosphorylation sites in cyan^20,21,58^, and a phosphorylation site with an unknown role in yellow^59^. The position of the RPA49 tWH domain from the Pol I EC is encircled and filled with transparent purple. N and C termini are labeled. **b**, RPA43 subunit and RRN3 are shown in cartoon representation, and the rest of Pol I is shown in surface representation, colored according to the subunit as in Fig. 1. The upper part of the RPA43 subunit from the Pol I–RRN3 structure (light blue) swings away (black arrow) from RRN3, compared with the RPA43 subunit, from the Pol I EC structure (dark blue, transparent). The lower part remains anchored to the core. The closest contact point between RPA43 and RRN3 (close-up view) is the location of the two residues (S199 and T200), which can carry an inactivating phosphorylation (cyan circle). The local resolution does not allow us to unambiguously identify the contacting residues from RPA43. RPA43 from the Pol I EC complex (dark blue transparent) would clash with the RRN3, and thus it swings by 5 Å away (white arrow) into the Pol I–RRN3 conformation.

Human RRN3 binds Pol I in a position similar to the one observed in yeast^26,27,29^. It contacts the stalk and residues 468–542 (dock) of subunit RPA1 (Fig. 5a). Superimposition with the Pol I EC suggests that the RPA49 tWH domain would clash with RRN3 (Fig. 5a). It has been suggested that the RPA49 tWH domain may help dislocate RRN3 in the transition from initiation to elongation^27,35^. Yet, a structure containing both RRN3 and A49 tWH is available from yeast^27^. In this structure, a larger portion of the RRN3 C terminus is ordered than in the human Pol I–RRN3 structure. In humans, RPA49 can interact with the C-terminus of RRN3 (ref. ^57^), which might also subsequently become ordered at a later stage of the transcription cycle.

In human Pol I, the main interaction site for RRN3 is the RPA43 subunit. The absence of a human subunit equivalent to yeast A14 introduces a hinge in the middle of the stalk-forming subunit. This allows the upper part of the stalk to swing away by ~5 Å upon RRN3 binding, while the bottom part remains anchored to the Pol I core (Fig. 5b). Binding of RRN3 to human Pol I relies on the phosphorylation status of RRN3 (refs. ^20–22,58,59^). The majority of residues that can be phosphorylated lie in disordered regions of RRN3 (Fig. 5a), which may become ordered upon interaction with SL1 or UBF. However, two residues (S199, T200) that have been shown to be a target of inactivating phosphorylation lie at the interface with subunit RPA43 (Fig. 5b, right). We speculate that phosphorylation at these two positions, adjacent to each other, may function as a phospho-switch and disrupt the interaction between RRN3 and RPA43, for example by changing the charge distribution in the interaction area (Fig. 5b, right). RRN3 also needs to be phosphorylated at several other sites to bind Pol I and to stimulate specific transcription initiation^60^. They are key for regulation of Pol I activity in response to proliferation signals^22^, nutrient availability^21^, or stress^20^. In addition to directly affecting the affinity between RRN3 and Pol I, some of these phosphorylations might also stimulate human Pol I activity by increasing the affinity of RRN3 to the accessory factors SL1 and UBF.

### Disease-associated mutations in Pol I

**Fig. 6:**
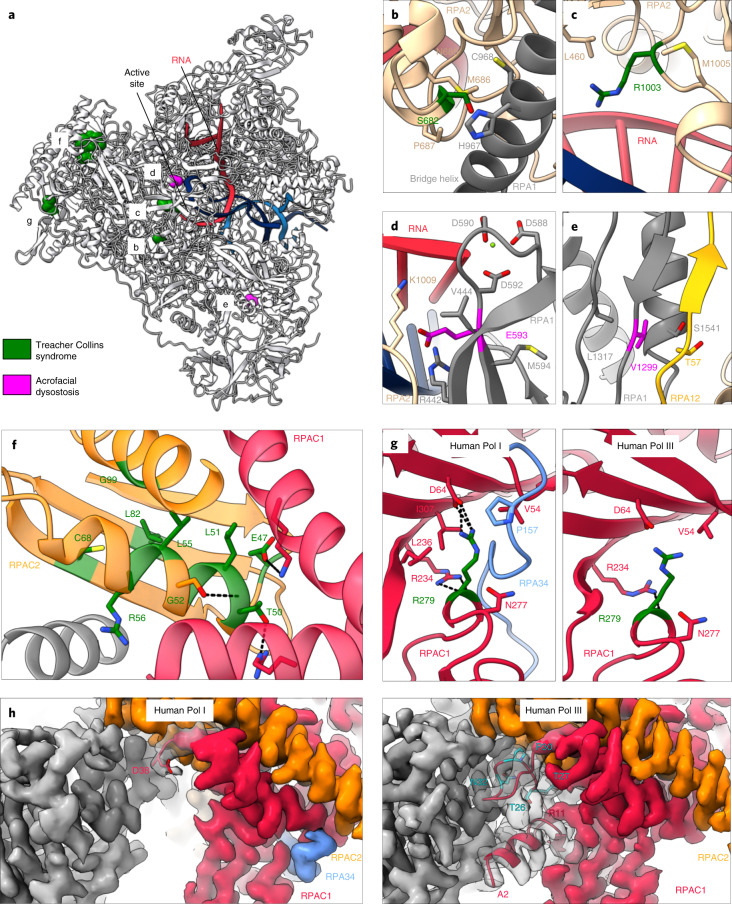
Disease-associated mutations of Pol I. **a**, Overview of the mutations affecting Pol I. The structure is shown in cartoon representation (gray), with nucleic acids marked in blue (DNA) and red (RNA). Disease-causing mutations for TCS (green) and AD (magenta) are shown in sphere representation. **b**–**g**, Close-up views of the residues affected by the mutations (stick representation). Other Pol I residues that can make contacts with the mutated one are also shown in stick representation. Putative hydrogen bonds are shown with black, dashed lines. **b**, S682 residue from RPA1. **c**, R1003 residue from RPA2. **d**, E593 residue from RPA1. Active-site residues found in close proximity to the E593 residue are shown in stick representation. **e**, V1299 residue from RPA1. **f**, Cluster of mutations in the RPAC2 subunit. Only residues affected by mutations and those that can form hydrogen bonds with them are shown for simplicity. **g**, RPAC1 residue R279 is shown in human Pol I (left panel) and in human Pol III (PDB: 7AE1 (ref. ^33^)) (right). **h**, Cryo-EM density corresponding to the core (gray) of the human Pol I (left) and human Pol III (right). Subunit RPAC1 is shown in red, RPAC2 in orange, and Pol I-specific RPA34 in blue. N-terminal residues are shown in cartoon representation together with their corresponding density in gray (transparent representation). The first visible residue in Pol I (D38) is shown in stick representation. Residues mutated in HLD found in the RPAC1 N-terminus (visible in Pol III) are shown in stick representation (cyan) (right).

Several mutations causing disorders known as ribosomopathies are found within Pol I itself^7^. They cluster in subunits RPAC1 and RPAC2, shared between Pol I and Pol III, as well as around the Pol I active site (Fig. 6a). Mutations within subunit RPA2 cause TCS, which is a craniofacial developmental disorder^9^. Residue S682 contacts the bridge helix (Fig. 6b), while residue R1003 lies within the hybrid binding region and interacts with the fork (Fig. 6c). Precise positioning of those elements throughout the transcription cycle is required for Pol I function and thus alterations in these regions could greatly impair Pol I activity. Mutations causing AD, another disease associated with craniofacial abnormalities, are found within the RPA1 subunit^8^. Residue E593 is found adjacent to the catalytic aspartate triad within the active site (Fig. 6d), and could interfere with nucleotide addition. Residue V1299 is located within the jaw domain, where it contacts the RPA12 linker (Fig. 6e). This interaction could be important for the correct insertion of the RPA12 C-terminal domain and thus could influence Pol I inactivation or backtracking. Many TCS-causing mutations are found in the RPAC2 subunit, which is important for core stability (Fig. 6f)^61,62^. Since this subunit is shared between Pol I and Pol III, it is possible that mutations in it affect both Pol I and Pol III. Another TCS-causing mutation, R279 in subunit RPAC1, is also shared between Pol I and Pol III. In human Pol III, the mutated residue R279 is exposed to solvent and does not make many contacts with other subunits (Fig. 6g, right)^33^. In human Pol I, on the contrary, R279 binds to the RPA34 extension that buries this residue (Fig. 6g, left). Using immunofluorescence and affinity pull-downs, it was shown that R279 mutation does not affect assembly of Pol III, but it influences the localization of Pol I to the nucleolus^63^. Therefore, the impaired nucleolar localization or assembly of Pol I presumably results in the TCS-causing effect of this mutation. Subunits RPAC1 and RPAC2, as well as Pol III-specific subunits, also harbor many mutations causing hypomyelinating leukodystrophy (HLD)^12,33,63^. Some of those mutations cluster in the N-terminus of the RPAC1 subunit (residues 26–32)^12^, which is fully ordered in human Pol III, making it an important site for interaction with other core subunits (Fig. 6h, right). However, the RPAC1 N terminus (1–37) is disordered in human Pol I (Fig. 6h, left). Thus, HLD-associated mutations in this region are less likely to impair the activity of Pol I. In line with our observations, it was shown that the mutation of one of those residues (N32) impairs the assembly of Pol III, but not Pol I^63^. Our structure suggests that HLD is likely to arise from perturbations to Pol III and not Pol I. On the contrary, TCS is more likely a result of Pol I malfunction. The high-resolution structure of human Pol I can help to explain the consequences of mutations on the molecular level and direct further studies of disease-associated mutations. Together with the structure of human Pol III^33^, it also enables distinguishing effects of mutations in subunits that are shared between Pol I and Pol III.

## Discussion

In this study, we present high-resolution structures of human Pol I in its elongating state at 2.7 Å resolution, bound to RRN3 at 3.1 Å resolution, and bound to an open DNA scaffold at 3.3 Å resolution. Despite the conservation of the general architecture of Pol I throughout the tree of life, several features distinguish human Pol I from yeast Pol I. First, we show that human Pol I can accommodate double-stranded RNA in the exit tunnel, which might increase the elongation speed^39^. In addition, the large funnel at the end of the RNA exit tunnel might assist the co-transcriptional folding of rRNA.

The exact subunit composition of human Pol I was previously unknown, as the human homolog of yeast stalk subunit A14 had not been identified. We now show that the human Pol I stalk is formed by a single subunit. In fact, a single-subunit stalk in Pol I is conserved throughout the tree of life, with the exception of certain fungi including yeast. Thus, we suggest that Pol I should be generally referred to as a 13-subunit complex.

The structures of Pol I–RRN3 and Pol I OC represent inactive states of human Pol I, with RPA12 being inserted into the funnel, an unfolded bridge helix and trigger loop, and a conserved (yet weakly bound) DNA-mimicking loop. Thus, critical elements that can regulate Pol I activity and render it inactive are also conserved between yeast and humans.

The structure of human Pol I also sheds light on the molecular basis of mutations that cause developmental disorders, such as TCS, AD, or HLD. By comparing the structure of Pol I with the human Pol III structure^33^, we can rationalize the observation made by Thiffault et al.^63^ suggesting that HLD and TLD might arise from perturbations in Pol III and Pol I, respectively, even though the causative mutations lie in subunits shared between both polymerases. Therefore, the human Pol I structure better explains the effect of disease-causing mutations and lays ground for further investigations of disease mechanisms.

So far, detailed structural insights into human Pol I have been lacking. Our study highlights the considerable differences between yeast and human Pol I. Human Pol I and its general transcription factors are well-established targets of anticancer drugs^3^. We are convinced that this high-resolution cryo-EM structure of human Pol I will aid the design of additional, highly specific human Pol I-specific inhibitors, further supporting efforts to combat cancer.

## Methods

### Endogenous tagging of RPAC1 with CRISPR–Cas9

The cell line with homozygously tagged RPAC1 generated in Girbig et al.^33^ was used for purification of the human Pol I. Briefly, HEK293T cells were transfected using polyethylenimine reagent in Opti-MEM I Reduced Serum Medium (Gibco) with a pSpCas9(BB)-2A-GFP (PX458) plasmid (Addgene plasmid no. 48138)^64^ containing a guide RNA (5′-ACTGAGCTTGGATGCTTCTG-3′) and a donor plasmid containing 700-bp homology arms and a RPAC1 tag synthesised by GenScript into pUC57-Mini plasmid. The C-terminal RPAC1 tag contained mCherry, Strep II, 6×His, and P2A peptide, followed by a blasticidin resistance gene and a SV40 termination signal. Cells were selected 5 days after transfection with 5 µg/mL blasticidin (Thermo Fisher Scientific). They were then seeded at low density, and single-cell colonies were picked and genotyped by PCR. A clone with correct tag integration was adapted to growth in suspension. For assessing the localization of the tagged complex, adherent cells were seeded to 20% confluency 2 days before imaging and were supplemented with 1 μg/mL live Hoechst dye. The medium was replaced with fresh medium immediately before imaging. Cells were imaged using a confocal microscope Olympus FV3000 using an UPLSAPO ×60 silicone objective. Hoechst staining was excited with 405 nm excitation laser and detected within emission range of 413–513 nm; mCherry was excited with 561 nm laser and detected within the range of 580–680 nm. Images were processed using Fiji^65^.

### Purification of human Pol I

Cells with tagged RPAC1 were grown in suspension in Expi293 Expression Medium (Thermo Fisher Scientific) up to 7 × 10^6^ cells/mL. They were collected by centrifugation, followed by washing with PBS and the pellets were flash-frozen for storage. Approximately 50 g of cell pellets was resuspended by stirring in lysis buffer (25 mM HEPES pH 7.5, 150 mM (NH_4_)_2_SO_4_, 5 mM MgCl_2_, 5% glycerol, 20 mM imidazole, 0.5% Triton X-100, and 2 mM β-mercaptoethanol in the presence of EDTA-free protease inhibitor cocktail (Roche) and benzonase (Sigma-Aldrich)). Cells were sonicated, centrifuged for 1 hour at 235,000*g* at 4 °C and filtered. The cleared lysate was applied to a 5 mL HisTrap HP column (GE Healthcare) and washed with Ni wash buffer containing 25 mM HEPES pH 7.5, 150 mM (NH_4_)_2_SO_4_, 5 mM MgCl_2_, 5% glycerol, 20 mM imidazole, and 2 mM β-mercaptoethanol. The complex was eluted with a 300 mM imidazole step. Eluted fractions were combined and incubated for 1 hour at 4 °C with 3 mL (50% bead slurry) of equilibrated Strep-Tactin agarose beads (IBA Lifesciences). Beads were applied to a gravity column, washed with buffer containing 25 mM HEPES pH 7.5, 200 mM CH_3_CO_2_K, 5 mM MgCl_2_, 5% glycerol, and 2 mM β-mercaptoethanol, and were eluted in 3 fractions with a buffer containing 20 mM biotin. Obtained fractions were pooled and applied to a 0.8 mL MiniQ 4.6 50 PE column (GE Healthcare). A gradient from 200 mM to 2 M CH_3_CO_2_K was used for elution, and Pol I eluted in two fractions at approximately 40 mS/cm and 50 mS/cm. The first peak was used for further studies. Pol I was concentrated on 100K spin concentrator (Merck Millipore) up to 0.7–1 mg/mL and buffer exchanged into the EM buffer containing 15 mM HEPES, pH 7.5, 80 mM (NH_4_)_2_SO_4_, 5 mM MgCl_2_, and 10 mM DTT. Quality of the complex was assessed with SDS–PAGE stained with Coomassie blue, and the identity of all subunits was confirmed with mass spectrometry. The obtained sample was directly used for grid preparation or flash-frozen and stored for in vitro assays.

### Purification of RRN3

N-terminal His-tagged human RRN3 was cloned into pETM11 plasmid (EMBL Protein Expression and Purification Core Facility). It was transformed into *Escherichia coli* LOBSTR expression strain (Kerafast). Cells were grown overnight at 18 °C in TB medium and expression was induced with 0.05 mM of IPTG at OD_260nm_ = 0.8–1.0. Pellets were collected by centrifugation and resuspended in lysis buffer (50 mM Tris-HCl pH 7.5, 200 mM NaCl, 10% glycerol, 10 mM imidazole, 2 mM β-mercaptoethanol in presence of Dnase 1 (Roche), EDTA-free protease inhibitor coctail (Roche) and lysozyme (Sigma-Aldrich)). Cells were disrupted using Microfluidizer Processor M-110L (Microfluidics), followed by centrifugation. The supernatant was incubated for 1 h with Ni-NTA agarose beads (Qiagen) at 4 °C. The agarose beads were then first washed with wash buffer 1 (50 mM Tris-HCl pH 7.5, 1 M NaCl, 10% glycerol, 40 mM imidazole, 2 mM β-mercaptoethanol and 5 mM ATP), followed by wash buffer 2 (50 mM Tris-HCl pH 7.5, 200 mM NaCl, 10% glycerol, 10 mM imidazole, 2 mM β-mercaptoethanol), and eluted with the elution buffer (50 mM Tris-HCl pH 7.5, 200 mM NaCl, 10% glycerol, 150 mM imidazole, 2 mM DTT). Eluted RRN3 was dialyzed in buffer A (20 mM Tris-HCl pH 7.5, 200 mM NaCl,2 mM DTT), and the N-terminal His tag was cleaved off by overnight incubation at 4 °C with TEV protease (EMBL Protein Expression and Purification Core Facility). The complex was then incubated for 30 min with the Ni-NTA beads (Qiagen) at 4 °C to capture the cleaved tag and the protease. The flow through was collected and applied to a MonoQ column (GE Healthcare). The RRN3 was eluted with a gradient over 10 column volumes of buffer B (20 mM Tris-HCl pH 7.5, 1 M NaCl, 2 mM DTT). The fractions containing RRN3 were concentrated using a 5-kDa cut-off concentrator (Corning) and injected on to a Superdex 200 increase 10/300GL size-exclusion column (GE Lifesciences) pre-equilibrated with the gel filtration buffer (25 mM Tris-HCl pH 7.5, 150 mM NaCl, 2 mM DTT). The fractions containing pure human RRN3 were concentrated up to 10–15 mg/mL and flash-frozen in liquid nitrogen for storage.

### Nucleic acid scaffold preparation

Nucleic acid oligonucleotides (Sigma-Aldrich, HPLC-grade) used included: template DNA:-5′ GTACTGAATTAGACAATGCTCTGTGGCTCTAGTACCATGAGCG-3′; nontemplate DNA: 5′-CGCTCATGGTACTAGGCTTCGGAGAAGTTGTCTAATTCAGTAC-3′, dimerizing RNA primer: 5′-UAUGCAUAACGCCACAGAG-3′ (used in transcription assay and for cryo-EM sample preparation), and single-stranded RNA primer: 5′-UCUGGUAUACGCCACAGAG-3′ (used in transcription assay). Template and nontemplate DNA at 100 μM in H_2_O were mixed and heated up to 95 °C for 2 minutes. They were immediately transferred to ice for 5 minutes of incubation and supplemented with 2× hybridization buffer (40 mM HEPES pH 7.5, 24 mM MgCl_2_, 200 mM NaCl, 20 mM DTT). RNA at 100 μM was heated on a 55 °C heating block for 1 minute and added to the DNA scaffold on ice. The resulting DNA–RNA mixture was brought to room temperature. For the Pol I OC complex preparation, steps with the RNA were omitted.

### In vitro transcription assay

The RNA primer was labeled at the 5′ terminus with [γ-^32^P]ATP using T4 PNK (New England Biolabs), and was PAGE purified. It was assembled with the DNA scaffold as outlined above. For the RNA extension assay, 2 pmol of the radioactively labeled nucleic acid scaffold was incubated with 3 pmol of Pol I for 10 min at room temperature. Subsequently, 0.2 mM of NTPs in the buffer containing 20 mM HEPES pH 7.5, 60 mM (NH_4_)_2_SO_4_, 10 mM MgSO_4_, and 10 mM DTT were added to the reaction mixture before incubation for 45 minutes at 37 °C. The reaction was stopped by addition of formamide. Samples were heated for 3 minutes at 95 °C and loaded on a denaturing 17% gel (17% acrylamide/bis 19:1, 8 M urea, TBE 1%). The radioactive products were recorded using phosphor-imaging screens (Fujifilm) for capturing digital images.

### Phylogenomic analysis

Reference proteomes for selected species (listed in the Supplementary Table 2) were downloaded from the UniProt website (version 2021_02)^66^. The phylogenetic tree of eukaryotic species was obtained from the NCBI Taxonomy Database^67^. Homologs for proteins of interest in this study were retrieved with the HHpred^68^ and the HMMER tool (v3.2.1)^69^, using individual protein sequences and domain families from the Pfam database (version 34.0)^70^. The following UniProt and Pfam identifiers were used for protein homology searches: A14 (UniProt: P50106, Pfam: PF08203), A43 (UniProt: P46669, Pfam: PF17875), ABC23 (UniProt: P20435, Pfam: PF01192), A135 (UniProt: P22138, Pfam: PF00562), and RPB4 (UniProt: P20433, Pfam: PF00562). The Pfam family for A14 (Pfam: PF08203) has been improved to include the *S. pombe* homologous protein, previously missing from the model. Paralogs for homologs of RPB4 subunit have been assigned according to their similarity to the *S. cerevisiae* protein (UniProt: P20433). For the A43 subunit, a new HMM model was created with HMMER^69^, based on the multiple sequence alignment, generated using the MUSCLE tool (v3.8)^71^, of A43 proteins in six species: *Homo sapiens*, *S. cerevisiae*, *Drosophila melanogaster*, *Arabidopsis thaliana*, *Dictyostelium discoideum*, and *S. pombe*. The Pfam family of the A43 OB domain (Pfam: PF17875) has been iterated to include missing domain annotations in higher eukaryotes. Structural insertions in A135 and ABC23 were manually assigned based on a multiple sequence alignment of full protein homologs generated using MUSCLE^71^. The Interactive Tree of Life (iTOL) online tool^72^ was used to visualize and annotate the phylogenetic tree and create the final figure. Sequence consensus score and occupancy score across the ABC23 subunit consensus sequence in Extended Data Fig. 3 was generated using Jalview^73^. Multiple sequence alignments in Extended Data Figs. 4, 5, and 8 were obtained using Clustal Omega^74^ and plotted using TeXshade^75^.

### Cryo-EM sample preparation

Freshly purified Pol I was used to prepare cryo-EM grids. For the Pol I EC sample, annealed DNA–RNA nucleic acid scaffold in 1.5 molar excess was added to the Pol I at 0.7 mg/mL. For the Pol I–RRN3–OC sample, Pol I at 0.85 mg/mL was mixed with RRN3 (diluted in EM buffer) in equimolar ratio and incubated on ice for 20 min prior to the addition of the DNA OC scaffold in 1.5 molar excess. Both samples were incubated for 30 minutes at room temperature and then were kept on ice until plunge freezing. Mesh 200, Cu R2/1 grids (Quantifoil) were plasma cleaned using a NanoClean plasma cleaner (Fischione Instruments, Model 1070) with a 75%–25% argon–oxygen mixture for 30 seconds. Grids were then plunge-frozen in liquid ethane using Vitrobot Mark IV (Thermo Fisher Scientific), set to 100% humidity and 15 °C. Then, 2.5 μL of sample was applied to the grid, and it was blotted using the following parameters: blot force 3, blot time 0 seconds, wait time 0 seconds.

### Data collection (Pol I EC)

The Pol I EC dataset was collected on a Titan Krios TEM operated at 300 keV (Thermo Fisher Scientific), equipped with a K3 direct detector (Gatan) and a Quantum energy filter (Gatan) using SerialEM^76^. Magnification of ×105,000, corresponding to a pixel size of 0.822 Å/pixel, was used. We recorded 10,053 movies in counting mode with 1.77 e^–^/Å^2^/frame over 38 frames with a defocus range of 1.0–2.5 μm.

### Data collection (Pol I–RRN3 and Pol I OC)

Grids with Pol I–RRN3–OC sample were pre-screened on FEI Talos Arctica microscope equipped with a Falcon III detector. A small dataset of 967 stacks was recorded with SerialEM^76^ at a magnification of ×92,000, corresponding to a pixel size of 1.566 Å/pixel. Exposure of 3.62 e^–^/Å^2^/frame over 12 frames was used, with a defocus range of 1.0–2.5 μm.

A high-resolution dataset was acquired as for the Pol I EC (Titan Krios TEM operated at 300 keV with magnification of ×105,000, corresponding to a pixel size of 0.822 Å/pixel), but 14,224 movies with 40 frames and exposure of 1.03 e^–^/Å^2^/frame were collected. A defocus range of 0.75–2.25 μm was used.

### Data processing (Pol I EC)

The overall processing pipeline for the Pol I EC is outlined in Extended Data Fig. 1. WARP 1.0.7W^77^ was used for the initial preprocessing of micrographs: frame alignment, contrast transfer function (CTF) estimation, and dose weighting. Next, 2,171,736 particles were picked with the network BoxNet2Mask_20180918 and extracted with a box size of 280 pixels. Particles were imported into CryoSPARC^78^ and subjected to 2D classification. We selected 20 out of 200 classes with clear 2D class averages showing secondary-structure features. No dimers were observed in 2D class averages. The 942,771 selected particles were subjected to ab initio classification. One out of two produced models resembled the yeast Pol I, and it was refined using Homogeneous Refinement (Legacy). The obtained map reached the overall resolution of 3.4 Å and was further used as a reference. Further processing was performed with RELION 3.1 (ref. ^79^). All micrographs were motion-corrected using RELION’s own algorithm according to MotionCor2 (ref. ^80^) and were CTF-corrected using Gctf^81^. Particles selected by WARP were re-extracted with a box size of 70 pixels (binned by a factor of 4). The reference obtained from cryoSPARC was appropriately resized using EMAN2 package^82^ and low-pass filtered to 40 Å in RELION^79^. The reference was used in a global 3D classification using T-parameter of 20. T-parameter = 20 was used in all 3D classification steps for both datasets. Three out of 8 classes that contained a complete Pol I map were selected, and 769,742 particles were re-extracted into a box size of 160 pixels (binned by a factor of 2) and 280 pixels (unbinned). Unbinned particles were CTF-refined and subjected to Bayesian polishing^83^, giving a map that resolved up to 2.8 Å. While the map reached high resolution, it had rather poor density in the upper clamp and stalk region. We fitted a human Pol I homology model, based on the yeast Pol I crystal structure (PDB: 4C3I)^14^, into the obtained map, and, using UCSF Chimera^84^, we created a general soft mask using the molmap command. Next, we performed masked 3D classification using the general soft mask. One of the two obtained classes with 198,822 particles had more high-resolution features throughout the map, and it was thus CTF-refined, polished, and CTF-refined a second time. The obtained map, denoted as Map A, has an overall resolution of 2.7 Å.

To improve the density in the stalk region, particles selected after the first global 3D classification (binned by a factor of 2), were subjected to masked 3D classification with a soft mask in the stalk region. In the discarded class, all subunits were present with at least partial density, but the map quality was overall lower, especially in the upper clamp and stalk regions. One of the two classes with 222,815 particles showed improved density in the region of interest and resolved up to 3.7 Å. Additional fuzzy density close to the stalk could also be observed at higher threshold levels. Guided by yeast elongating Pol I structure (PDB: 5M64)^25^, we fitted a homology model for the RPA49 tWH domain. A soft mask covering the stalk and the tWH of RPA49 was used for another masked 3D classification. A class containing 37,180 particles showed improved density in the region and had an overall resolution of 3.9 Å. The same strategy of CTF refinement and particle polishing was employed and the resolution improved up to 3.0 Å, giving Map B. Using the fitted homology model of human Pol I, two soft masks were created using Chimera^84^: the first covered the upper clamp, stalk, tWH of RPA49, and the nucleic acid scaffold, and the second contained the core of the Pol I and the heterodimer. Those masks were used for multibody refinement in RELION^85^, which yielded partial maps B1 and B2. The resolution of all maps was obtained with RELION’s post-processing tool based on the gold-standard Fourier shell correlation (FSC) using the 0.143 cut-off criterion^86^. The local resolution range was estimated using RELION’s local resolution tool. Map C is a composite map obtained using the “combine_focused_maps” command implemented in the Phenix 1.18 (ref. ^87^), supplied with the Map A, B, and B1. Map A was sharpened with the LocalDeblur tool^88^ from Scipion^89^, while maps B, B1, B2, and C were sharpened with the Autosharpen tool from Phenix^90^. RStudio^91^ was used to plot the FSC curves.

### Data processing (Pol I–RRN3 and Pol I OC)

The processing pipeline for the Pol I–RRN3 and Pol I OC dataset is outlined in Extended Data Fig. 6. First, a small dataset of 967 micrographs was collected on a FEI Talos Arctica. WARP 1.0.7W^77^ was used for the frame alignment, CTF estimation, and dose weighting of the micrographs. Using the standard network, 375,673 particles were picked in WARP and extracted into a 220-pixel box. Particles were imported into cryoSPARC^78^ and 2D classified. Ten out of 50 classes were selected, and 161,124 particles were subjected to ab initio reconstruction, asking for two classes. One obtained class resembled Pol I only, while the other, with 45,335 particles, showed additional density next to the stalk where the RRN3 was expected to bind. It was selected and refined with the homogeneous refinement tool yielding a map resolving up to 7 Å, which was further used as a reference. To obtain high-resolution data for the Pol I–RRN3–OC sample, we collected a dataset on the Titan Krios TEM. As for Pol I EC, WARP^77^ was used for initial micrograph preprocessing and particle picking. 2,628,144 particles were picked and extracted into a 288-pixel box. The dataset was further processed with RELION 3.1 (ref. ^79^). Micrographs were preprocessed using Relion’s own implementation of MotionCor2 (ref. ^80^) and Gctf^81^, same as for the Pol I EC dataset. Particles were extracted with a 72-pixel box (binned by a factor of 4) and split into 5 subsets for faster processing. Each subset was subjected to a global 3D classification with 8 classes using the reference obtained from the FEI Talos Arctica adjusted for the box and pixel size using the EMAN2 package^82^. In each subset, classes containing complete Pol I were selected, and those particles were re-extracted into the box of 144 pixels (binned by a factor of 2). Using Chimera^84^, the structure of the human Pol I EC was fitted into the map. The structure of the yeast Pol I with RRN3 (PDB: 6RQT)^27^ was matched to it to guide the fitting of the homology model for the human RRN3. A soft mask covering the stalk and the RRN3 was created, and it was used for masked 3D classification. In each subset, the class that contained a more defined signal in the region of interest was selected. Those particles from all subsets were pooled, giving 345,425 particles in total. The resulting intermediate map resolved up to 3.3 Å while data were binned by a factor of 2. Another 3D classification with a soft mask on RRN3 only allowed splitting the dataset into two classes: first (referred to as Pol I–RRN3) with 169,513 particles showed signal for the RRN3; and second (referred to as Pol I OC) with 175,912 particles had signal for the nucleic acid, but not for the RRN3. The Pol I–RRN3 class resolved up to 3.4 Å, while the Pol I OC reached 3.6 Å. Both of the classes were unbinned, CTF-refined and polished with the same strategy as for the Pol I EC dataset. The resolution improved up to 3.2 Å for Pol I–RRN3 and to 3.3 Å for Pol I OC, and the obtained maps are denoted as Map D and F, respectively. Still, the density corresponding to the upper clamp and the RRN3 in the Map D appeared streaky, possibly due to the clamp movement which distorts the signal. Thus, we applied focused refinement strategy to the intermediate map binned by factor of two^85^. A soft mask covering the upper clamp and the RRN3 was applied from the fourteenth iteration of the 3D refinement. The obtained map was used as a reference in 3D classification with a mask covering the stalk and the RRN3. The obtained class with density for RRN3 contained 260,363 particles, which were re-extracted into a 288-pixel box.The second round of focused refinement was applied to the unbinned particles in the same manner as previously described. The map was then CTF-refined and polished, resulting in a map with the overall resolution of 3.1 Å and improved density for the upper clamp and the RRN3. It was denoted as Map E. Map F, corresponding to the Pol I OC, was later subjected to the masked classification with a soft mask covering the nucleic acids. A class with improved density corresponding to the DNA scaffold included 164,436 particles, and, after CTF refinement and polishing, it resolved up to 3.3 Å giving Map G. Maps D, E, F, and G were sharpened with LocalDeblur^88^ and LocScale^92^ implemented within CCP-EM^93^. RStudio^91^ was used to plot the FSC curves. Effects of directional resolution anisotropy were assessed with the 3DFSC program^53^.

### Model building and refinement

A homology model based on the yeast Pol I crystal structure (PDB: 4C3I)^14^ was created using: PDB 7AE1 (ref. ^33^) for the subunits shared between Pol I and Pol III; homology models from the SWISS-MODEL Repository^94^ for the subunits RPA1, RPA2, RPA12, and RPA34; and homology models obtained from Phyre2^95^ for RPA43 and RPA49 subunits. PSIPRED^96^ was used to model secondary-structure elements not present in homology models. Subunits were aligned using UCSF Chimera^84^, and the model was rigid-body fitted into Map A. The nucleic acid scaffold from PDB 6HLR^97^ was fitted into the density within the cleft. The resulting model was further refined using COOT 0.9 (ref. ^98^). First, each chain was fitted as a rigid body into the density, and then ProSMART restraints^99^ were generated and used in the refinement to improve the fit. The model was subsequently manually rebuilt and refined in COOT. To build the downstream DNA, PDB 5M5X^25^ was initially fitted for guidance and then adjusted to fit the cryo-EM density. To build the double-stranded RNA in the RNA exit tunnel, a perfect RNA double-stranded helix (A-form) was fitted and then refined to match the density. The sequence of the nucleic acids was manually mutated to match the used scaffold. Stalk, tWH domain of RPA49, and the RNA in the exit tunnel were built using Map B1. Tracing of the linker of the RPA49 subunit was possible thanks to Map B and was partially aided by the yeast Pol I structure from PDB 6RQT^27^. The structure was iteratively refined using the real-space refinement tool^100^ from Phenix 1.13 (ref. ^101^) against Map A with secondary-structure restraints turned on. For the two final rounds of the refinement, Map C was used to account for the regions which were more poorly resolved in the Map A.

For building of the Pol I–RRN3 structure, Pol I EC was rigid-body fitted into the Map D using Chimera^84^. The nucleic acid scaffold, as well as the tWH domain of RPA49, were removed. The homology model for the RRN3 was generated using Phyre2^95^ and fitted into the density next to the stalk guided by the structure of yeast Pol I with RRN3 (PDB 6RQT)^27^. The structure for the RPA12 subunit, which included the C-terminal domain, was taken from the SWISS-MODEL Repository^94^ and fitted into the model. In COOT, ProSMART restraints^99^ were generated for all subunits, and they were refined into the map. For RPA1 and RPA2, subunits were split along the hinges of the clamp to allow more accurate fitting into the map. The upper clamp and active site were extensively rebuilt manually and refined in COOT^98^. At later stages of the building, Map E was used for the stalk and RRN3 building. Sharpening of the Map D and Map E using LocScale^92^ helped reveal detailed features. Into the extra density inside the DNA-binding cleft, a relevant part of the RPA1 subunit homology model from Phyre2 (ref. ^95^) was fitted using Map E and manually adjusted. This section was removed from the final model due to poor quality of the map, which did not allow register assignment. The fit of the model into the cryo-EM density was assessed in detail, and where necessary it was truncated or extended. The structure was refined against Map E using real-space refinement in Phenix 1.13 (ref. ^101^).

To obtain the model for the Pol I OC, the Pol I–RRN3 structure was fitted into the cryo-EM density. The RRN3 structure was removed. Nucleic acid scaffold from the PDB 5M5W^25^ was rigid-body fitted into the visible density inside the cleft using Map G. We truncated the nucleic acids and left only the downstream double-stranded portion of the template. The sequence was manually mutated to match the template used. The structure was manually adjusted and refined in COOT^98^. It was refined against Map F using real-space refinement tool from Phenix 1.13.

Refinement statistics reported within Table 1 were obtained by the MolProbity comprehensive validation tool^102^ implemented within Phenix 1.13 (ref. ^101^). The charge distribution presented in Fig. 2. was calculated using the APBS software^103^. Figures were prepared using ChimeraX^104^.

### Reporting Summary

Further information on research design is available in the Nature Research Reporting Summary linked to this article.

## Online content

Any methods, additional references, Nature Research reporting summaries, source data, extended data, supplementary information, acknowledgements, peer review information; details of author contributions and competing interests; and statements of data and code availability are available at 10.1038/s41594-021-00693-4.

## Supplementary information

Supplementary Information Supplementary Tables 1 and 2.

Reporting Summary

Peer Review Information

Supplementary Data 1 Sequence alignment of proteins homologous to human RPA2 subunit and of proteins homologous to human RPABC2 subunit. 2 FASTA files.

## Data Availability

Cryo-EM maps obtained within this study have been deposited to the EMDB database under following accession codes: EMD-12795 (Maps A, B, B1, B2, and C), EMD-12796 (Maps D and E), and EMD-12797 (Maps F and G). The coordinates of the atomic models have been deposited to the PDB with the following accession codes: 7OB9 (Pol I EC), 7OBA (Pol I–RRN3), and 7OBB (Pol I OC). Datasets from PDB used in this study include: 4C3I, 4C3J, 7AE1, 5M5X, 5M64, 6LHR, 6RQT, 6RUO, and 5FLM. Source data are provided with this paper.

## Source data

Source Data Fig. 1 Unprocessed microscopy images (Fig. 1a), Commassie stained SDS–PAGE gel (Fig. 1b) and blot for transcription assay (Fig. 1c).
Source Data Extended Data Fig. 2 Unprocessed blot for transcription assay (Extended Data Fig. 2b)

## Code availability

Code and further instructions for the phylogenomic analysis are available at: https://github.com/bateman-research/domain-phylo. Source data are provided with this paper.
