# Supplementary material for: Cryo-EM structures of human RNA polymerase I
Source: Nat Struct Mol Biol. 2021 Dec 9;28(12):997–1008. doi: 10.1038/s41594-021-00693-4 (PMC8660638; doi:10.1038/s41594-021-00693-4)
Supplement: Supplementary file 1 — Supplementary Tables 1 and 2. [file 41594_2021_693_MOESM1_ESM.pdf]

nature portfolio
https://doi.org/10.1038/s41594-021-00693-4

Supplementary information

Cryo-EM structures of human RNA polymerase I

In the format provided by the authors and unedited

Supplementary Information

Cryo-EM structures of human RNA polymerase I

Agata D. Misiaszek, Mathias Girbig, Helga Grötsch, Florence Baudin, Brice Murciano, Aleix Lafita, Christoph W. Müller

Supplementary Table 1: Mass spectrometry assessment of the purified Pol I complex.

Statistics for the highest scoring hit used for the identification of the subunit present in the protein band as visualized by coomassie stain in Fig. 1b.

Pol I subunit	Gene name	Number of unique peptides	Sequence coverage [%]	Max score
RPA1	*POLR1A*	246	89.9	222
RPA2	*POLR1B*	93	51.8	121
RPAC1	*POLR1C*	38	79.9	177
RPA49	*POLR1E*	55	67.4	137
RPA43	*TWISTNB*	4	18.0	95
RPA34	*CD3EAP*	8	10.8	70
RPABC1	*POLR2E*	40	79.6	144
RPABC3	*POLR2H*	20	78.8	183
RPABC2	*POLR2F*	4	14.8	71
RPA12	*ZNRD1*	14	63.0	82
RPAC2	*POLR1D*	16	63.4	102
RPABC4	*POLR2K*	5	45.8	82
RPABC5	*POLR2L*	2	29.4	64

Supplementary Table 2: Phylogenomic analysis of the stalk subunits and core contacting the stalk.

Listed are analyzed species, their proteome identifiers and UniProt identifiers for homologs of RPA43, A14 (*S. cerevisiae* smaller stalk subunit), RPABC2, RPA2 and RPB4 (Pol II smaller stalk subunit). Homologs were retrieved with the HMMER tool[1] as described in the Methods details. See Extended Data Fig. 3A for the corresponding phylogenomic tree.

Species	Proteome ID	RPA43	A14	RPABC2	RPA2	RPB4
Allomyces macrogynus	UP000054350	A0A0L0SVD6		A0A0L0SPJ3	A0A0L0T0Q0	A0A0L0SGA1
Anopheles gambiae	UP000007062	Q7PV20		Q7Q6F5	Q7QG74	F5HMV9
Arabidopsis thaliana	UP000006548	Q8H1F8		Q9SJ96	F4I366	O48890
Aspergillus flavus	UP000001875	B8NJ07		B8N1E2	B8N4K6	B8N071
Aspergillus nidulans	UP000000560	Q5ARH8		Q5B9N3	Q5B697	C8VJ36
Aspergillus niger	UP000006706	A2QYA8		A2Q8H6	A2QVK7	A2QEL2
Caenorhabditis briggsae	UP000008549	A8XP74		A8WSV7	A8XVM2	A8XH66
Caenorhabditis elegans	UP000001940	P34398		Q17684	Q27493	O02092
Candida albicans	UP000000559	A0A1D8PS34	Q5A4Y4	Q59QT0	A0A1D8PQM4	A0A1D8PH80
Canis lupus	UP000002254	F1PS84		A0A5F4CKL9	E2REX8	A0A5F4D7H3
Chaetomium thermophilum	UP000008066	G0SAD7		G0SHB3	G0SBR6	G0S0I8
Cryptosporidium parvum	UP000006726	Q5CSR5		Q5CXV6	Q5CSG7	Q5CU40
Cyanidioschyzon merolae	UP000007014	M1UPT2		M1VBK5	M1UWS4	M1VIP7
Danio rerio	UP000000437	Q6PHG8		A0A2R8PUZ9	B8JKD7	Q6DRG4
Dictyostelium discoideum	UP000002195	Q55FA4		Q54FA8	Q54BM1	Q54S04
Drechslerella stenobrocha	UP000024837	W7HNZ1		W7HVC9	W7I2Q7	W7HMD0
Drosophila melanogaster	UP000000803	Q9VM72		Q24320	P20028	Q9VEA5
Eremothecium gossypii	UP000000591	Q759A0	Q750W5	Q758N4	Q75DS1	Q75BM1
Felis catus	UP000011712	M3X5H4		A0A337SAL0	A0A337SSK7	M3WUR6
Gallus gallus	UP000000539	F1NJS0		Q9PT88	A0A1D5PW04	F1P2S1
Giardia intestinalis	UP000001548	A8BD91		E2RTN0	A8B827	A8BLT8
Homo sapiens	UP000005640	Q3B726		P61218	Q9H9Y6	O15514

Supplementary Table 2. (continued)

Leishmania major	UP000000542			Q4Q3B6	Q4QD43	E9ADP1
Mus musculus	UP000000589	Q78WZ7		P61219	P70700	Q9D7M8
Nannizzia gypsea	UP000002669	E4V032		E4V011	E4V6W4	E4UVA2
Naumovozyma dairenensis	UP000000689	G0WBT5	G0WGQ4	G0WHC7	G0WEC6	G0W378
Neocallimastix californiae	UP000193920	A0A1Y2FPE5		A0A1Y2E9Y1	A0A1Y2ER36	A0A1Y2FCK7
Neurospora crassa	UP000001805	Q1K5X0		A7UVS4	O74633	Q7SCW6
Oryza sativa	UP000007015	B8BM96		A2YL15	B8BHP0	A2X046
Pan troglodytes	UP000002277	H2QU84		A0A2I3R9X3	A0A2I3S2L7	H2QIP4
Physcomitrium patens	UP000006727	A0A2K1JED6		A0A2K1J8Y4	A0A2K1KJQ6	A9SKY3
Piptocephalis cylindrospora	UP000267251				A0A4P9Y8I8	A0A4P9Y6N3
Plasmodium falciparum	UP000001450	Q8I662		O77315	Q8II17	O96150
Pseudogymnoascus verrucosus	UP000091956	A0A1B8GXM5		A0A1B8GXU1	A0A1B8GIT1	A0A1B8G7S2
Rattus norvegicus	UP000002494	D4ADH7		O88828	O54888	D4A259
Rhizophagus irregularis	UP000236242	A0A2H5U221		A0A2H5SDC3	A0A2H5U9I3	A0A2H5RL53
Rhodotorula toruloides	UP000199069	A0A0K3CR30		A0A0K3C5S6	A0A0K3CA30	A0A0K3C489
Saccharomyces cerevisiae	UP000002311	P46669	P50106	P20435	P22138	P20433
Saccharomyces eubayanus	UP000050240	A0A0L8RBR2	A0A0L8RN62	A0A0L8RAJ0	A0A0L8RA78	A0A0L8RGH1
Schizosaccharomyces pombe	UP000002485	O43036	Q9P7P1	P36595	Q9P7X8	O74825
Sporisorium reilianum	UP000239563	A0A2N8U9J2		A0A2N8UHS9	A0A2N8U8X1	A0A2N8UKJ6
Takifugu rubripes	UP000005226	A0A3B5KGN9		A0A3B5KPZ4	H2SUR9	H2SFV1
Thalassiosira pseudonana	UP000001449			B8C3F5	B8CA95	B8CC64
Tuber borchii	UP000244722	A0A2T6ZK22		A0A2T6ZXD7	A0A2T6ZIG0	A0A2T7A3H4
Vitis vinifera	UP000009183	E0CPL7		E0CTZ1	F6HVC4	A5APH2
Wallemia ichthyophaga	UP000014064	R9AFX6		R9AGG3	R9AAU1	R9AGB7
Xylona heveae	UP000076632	A0A165I386		A0A165ILI2	A0A165GNP8	A0A165HMX8
Zea mays	UP000007305	B8A0V9		B6SI57	A0A1D6KRW8	B6SLK8

Description of Additional Supplementary Data

Supplementary Data 1. Sequence alignment of proteins homologous to human RPA2 subunit. Fasta file.

Supplementary Data 2. Sequence alignment of proteins homologous to human RPABC2 subunit. Fasta file.

Supplementary References

1. Eddy, S. R. Accelerated profile HMM searches. *PLoS Comput. Biol.* **7**, 1002195 (2011).
