# Supplementary material for: Cryo-EM structures of human RNA polymerase I
Source: Nat Struct Mol Biol. 2021 Dec 9;28(12):997–1008. doi: 10.1038/s41594-021-00693-4 (PMC8660638; doi:10.1038/s41594-021-00693-4)
Supplement: Supplementary file 2 — Reporting Summary [file 41594_2021_693_MOESM2_ESM.pdf]

nature research

Corresponding author(s): Christoph W Müller

Last updated by author(s): Oct 15, 2021

Reporting Summary

Nature Research wishes to improve the reproducibility of the work that we publish. This form provides structure for consistency and transparency in reporting. For further information on Nature Research policies, see our Editorial Policies and the Editorial Policy Checklist.

Statistics

For all statistical analyses, confirm that the following items are present in the figure legend, table legend, main text, or Methods section.

n/a	Confirmed	
☐	☒	The exact sample size (*n*) for each experimental group/condition, given as a discrete number and unit of measurement
☐	☒	A statement on whether measurements were taken from distinct samples or whether the same sample was measured repeatedly
☒	☐	The statistical test(s) used AND whether they are one- or two-sided<br>*Only common tests should be described solely by name; describe more complex techniques in the Methods section.*
☒	☐	A description of all covariates tested
☒	☐	A description of any assumptions or corrections, such as tests of normality and adjustment for multiple comparisons
☒	☐	A full description of the statistical parameters including central tendency (e.g. means) or other basic estimates (e.g. regression coefficient) AND variation (e.g. standard deviation) or associated estimates of uncertainty (e.g. confidence intervals)
☒	☐	For null hypothesis testing, the test statistic (e.g. *F*, *t*, *r*) with confidence intervals, effect sizes, degrees of freedom and *P* value noted<br>*Give P values as exact values whenever suitable.*
☒	☐	For Bayesian analysis, information on the choice of priors and Markov chain Monte Carlo settings
☒	☐	For hierarchical and complex designs, identification of the appropriate level for tests and full reporting of outcomes
☒	☐	Estimates of effect sizes (e.g. Cohen's *d*, Pearson's *r*), indicating how they were calculated

Our web collection on statistics for biologists contains articles on many of the points above.

Software and code

Policy information about availability of computer code

Data collection	SerialEM 3.8.0 beta
Data analysis	Warp 1.0.7W, RELION 3.1, cryoSPARC 2.15.0, EMAN2 2.22, LocalDeblur v2.0, LocScale v0.1, UCSF Chimera 1.15, UCSF ChimeraX 1.2, Coot 0.8.9.1, PHENIX 1.13, PHENIX 1.18, Scipion 2.0, Phyre2, 3DFSC 3.0, TeXshade 1.25, HHpred 3.2.0, PSIPRED 4.0, Jalview 2.11.0, R 3.5.3, RStudio 1.1.456, UniProt 2021_02, HMMER 3.2.1, Pfam database 34.0, iTOL v6, MUSCLE v3.8, Clustal Omega 1.2.2, Fiji 1.52n, APBS 3.2.1, MolProbity 4.5.1. Custom code used for phylogenetic analysis is available at https://github.com/bateman-research/domain-phylo.

For manuscripts utilizing custom algorithms or software that are central to the research but not yet described in published literature, software must be made available to editors and reviewers. We strongly encourage code deposition in a community repository (e.g. GitHub). See the Nature Research guidelines for submitting code & software for further information.

Data

Policy information about availability of data

All manuscripts must include a data availability statement. This statement should provide the following information, where applicable:

- Accession codes, unique identifiers, or web links for publicly available datasets
- A list of figures that have associated raw data
- A description of any restrictions on data availability

Cryo-EM maps of human RNA polymerase I have been deposited to the Electron Microscopy Data Bank (EMDB) database under following accession codes: EMD-12795 (Map A, B, B1, B2 and C), EMD-12796 (Map D and E) and EMD-12797 (Map F and G). The atomic models coordinates have been deposited to the Protein Data Bank (PDB) with the following accession codes: 7OB9 (Pol I EC), 7OBA (Pol I-RRN3) and 7OBB (Pol I OC).
Datasets from PDB used in this study include: 4C3I, 4C3J, 7AE1, 5M5X, 5M64, 6LHR, 6RQT, 6RUO, 5FLM.

Field-specific reporting

Please select the one below that is the best fit for your research. If you are not sure, read the appropriate sections before making your selection.

☒ Life sciences ☐ Behavioural & social sciences ☐ Ecological, evolutionary & environmental sciences

For a reference copy of the document with all sections, see nature.com/documents/nr-reporting-summary-flat.pdf

Life sciences study design

All studies must disclose on these points even when the disclosure is negative.

Sample size	Cryo-EM data was collected on two independently prepared samples. Numbers of collected micrographs and picked particles are detailed in the methods section. Collected sample size was dependent on the available microscope time and was sufficient to reach high resolution.
Data exclusions	Collected micrographs from both Pol I EC and Pol I OC / Pol I - RRN3 datasets were excluded based on pre-established criteria and on the fly calculated values provided by Warp 1.0.7W: 1) an estimated resolution worse than 6.0 Å (for the Pol I EC sample) or 8.0 Å (for the Pol I - RRN3 and Pol I OC sample) based on CTF fitting, 2) an average motion per frame in the first third of the movie larger than 2.0 Å (for the Pol I EC sample) or 5.0 Å (for the Pol I - RRN3 and Pol I OC sample) 3) Astigmatism higher than 3.0 σ. Particle images were sorted using RELION as described in methods section to achieve high-resolution 3D reconstructions and to resolve sample- and conformational heterogeneity.
Replication	Cryo-EM data was recorded once for each prepared sample. Protein purification was replicated independently 15 times with one failed attempt. Biochemical experiment from Fig. 1c was replicated 3 times, while the assay from Extended Data Fig.2b was not replicated due to low availability of material.
Randomization	Not relevant to the study - no experimental groups were used. Prepared samples were independent from each other.
Blinding	Investigators were not blinded during data acquisition and analysis since prior knowledge of the sample is needed for proper data collection and analysis, i.e. visual confirmation of the sample appearance and distribution during data collection or selection of the appropriate classes during data processing.

Reporting for specific materials, systems and methods

We require information from authors about some types of materials, experimental systems and methods used in many studies. Here, indicate whether each material, system or method listed is relevant to your study. If you are not sure if a list item applies to your research, read the appropriate section before selecting a response.

Materials & experimental systems

n/a	Involved in the study	
☒	☐	Antibodies
☐	☒	Eukaryotic cell lines
☒	☐	Palaeontology and archaeology
☒	☐	Animals and other organisms
☒	☐	Human research participants
☒	☐	Clinical data
☒	☐	Dual use research of concern

Methods

n/a	Involved in the study	
☒	☐	ChIP-seq
☒	☐	Flow cytometry
☒	☐	MRI-based neuroimaging

Eukaryotic cell lines

Policy information about cell lines

Cell line source(s)	HEK293T cells (human) were obtained from ThermoFisher.
Authentication	No cell-line authentication method was used.
Mycoplasma contamination	All cell lines tested negative for mycoplasma contamination.
Commonly misidentified lines (See ICLAC register)	No commonly misidentified cell lines were used.
