# Supplementary figures and images for: Cryo-EM structures of human RNA polymerase I
Source: Nat Struct Mol Biol. 2021 Dec 9;28(12):997–1008. doi: 10.1038/s41594-021-00693-4 (PMC8660638; doi:10.1038/s41594-021-00693-4)

Fig. 1a – source data

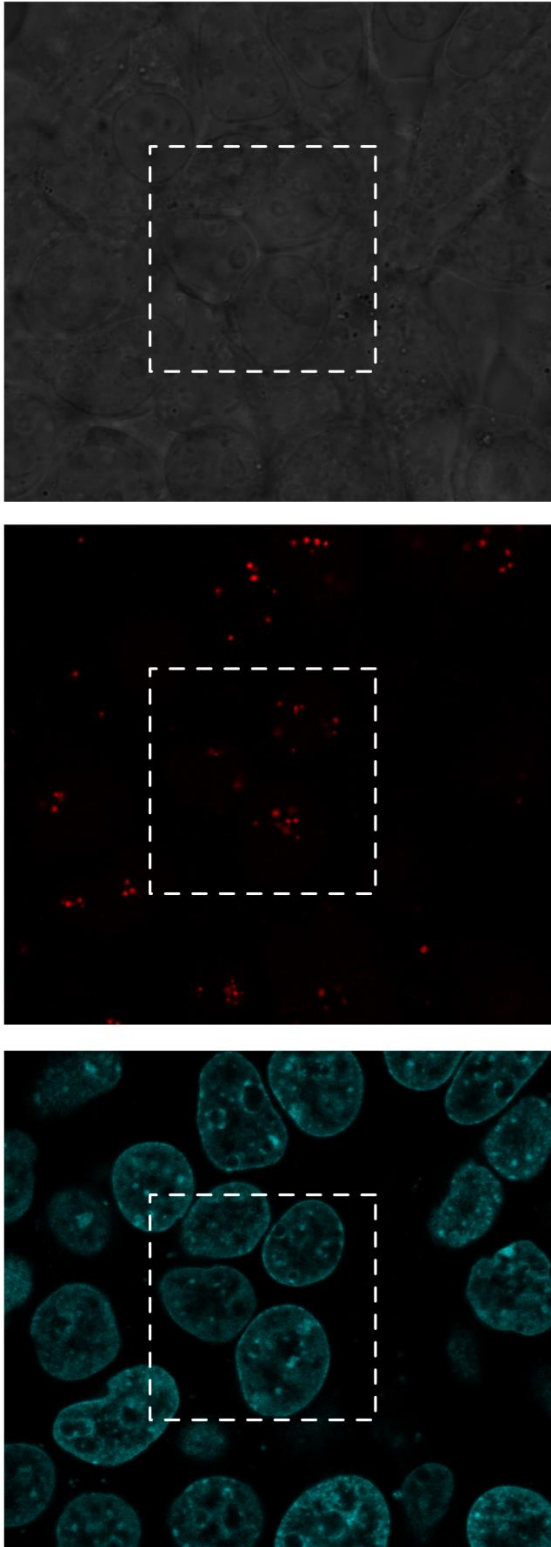

Fig. 1b – source data

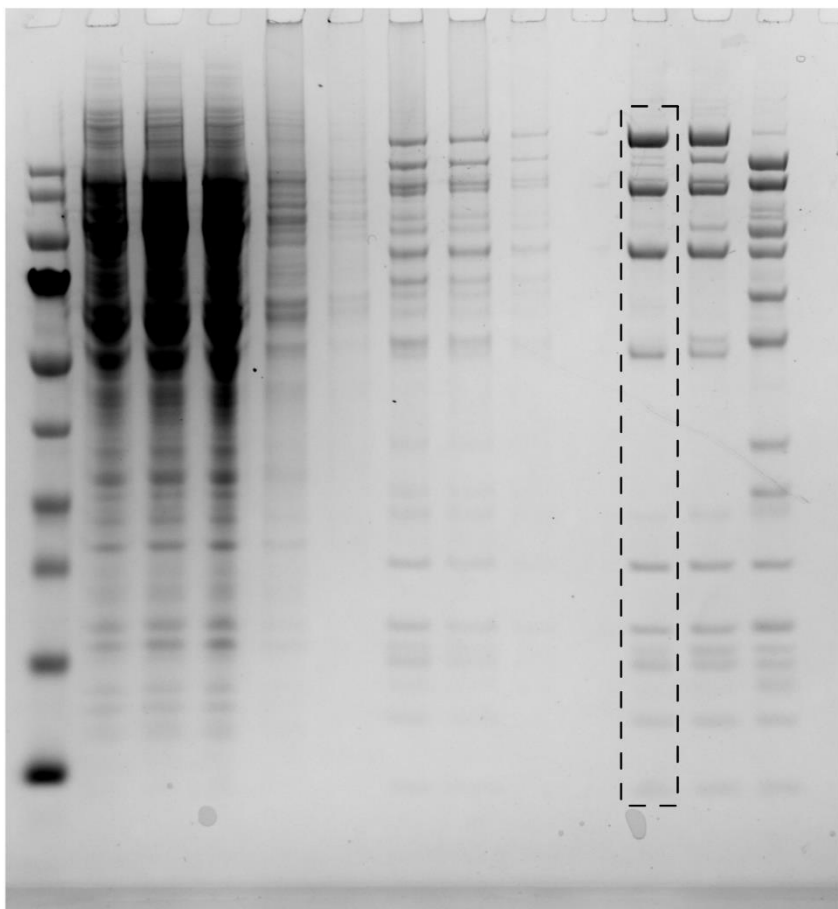

Fig. 1c – source data

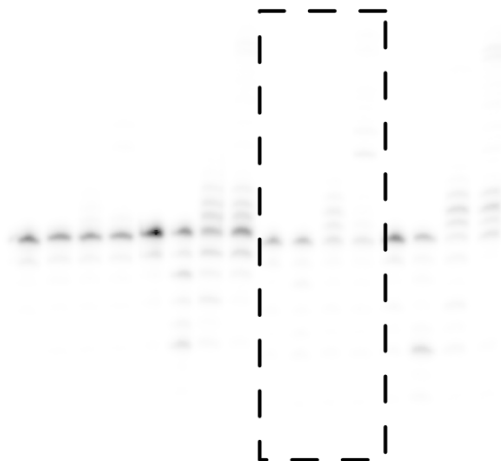

Supplement: Source Data Fig. 1 — Unprocessed microscopy images (Fig. 1a), Commassie stained SDS–PAGE gel (Fig. 1b) and blot for transcription assay (Fig. 1c). [file 41594_2021_693_MOESM5_ESM.pdf]

Extended Data Fig. 2b – source data

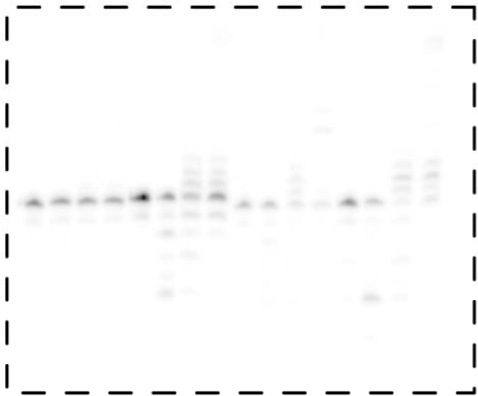

100 200 300 400 500 600 700 800 900 1000

Supplement: Source Data Extended Data Fig. 2 — Unprocessed blot for transcription assay (Extended Data Fig. 2b) [file 41594_2021_693_MOESM6_ESM.pdf]
