# Supplementary material for: Cryo-EM structures of human RNA polymerase I
Source: Nat Struct Mol Biol. 2021 Dec 9;28(12):997–1008. doi: 10.1038/s41594-021-00693-4 (PMC8660638; doi:10.1038/s41594-021-00693-4)
Supplement: Supplementary file 3 — Peer Review Information [file 41594_2021_693_MOESM3_ESM.pdf]

natureresearch

Peer Review Information

Journal: Nature Structural and Molecular Biology
Manuscript Title: Cryo-EM structures of human RNA polymerase I
Corresponding author name(s): Dr Christoph Müller

Reviewer Comments & Decisions:

Decision Letter, initial version:

5th Jul 2021

Dear Dr. Muller,

Thank you again for submitting your manuscript "Cryo-EM structures of human RNA polymerase I". We now have comments (below) from the 2 reviewers who evaluated your paper. In light of those reports, we remain interested in your study and would like to see your response to the comments of the referees, in the form of a revised manuscript.

You will see that both referees appreciate the results and find the conclusions timely and of wide interest. There are, however, some concerns and suggestions that should be addressed in a revision. Specifically, Reviewer #2 suggests that the structure may represent a paused rather than a canonical elongation complex, which needs to be further explored. Additionally, the analysis should include more comparison with previous published structures, as well as addressing the other referee comments. Please do not hesitate to contact me directly if you would like to discuss these issues further.

Please be sure to address and respond to all concerns of the referees in full in a point-by-point response and highlight all changes in the revised manuscript text file. If you have comments that are intended for editors only, please include those in a separate cover letter.

We are committed to providing a fair and constructive peer-review process. Do not hesitate to contact us if there are specific requests from the reviewers that you believe are technically impossible or unlikely to yield a meaningful outcome.

We expect to see your revised manuscript within 6 weeks. If you cannot send it within this time, please contact us to discuss an extension; we would still consider your revision, provided that no

similar work has been accepted for publication at NSMB or published elsewhere.

As you already know, we put great emphasis on ensuring that the methods and statistics reported in our papers are correct and accurate. As such, if there are any changes that should be reported, please submit an updated version of the Reporting Summary along with your revision.

Please follow the links below to download these files:

Reporting Summary:
https://www.nature.com/documents/nr-reporting-summary.pdf

Please note that the form is a dynamic 'smart pdf' and must therefore be downloaded and completed in Adobe Reader.

When submitting the revised version of your manuscript, please pay close attention to our href="https://www.nature.com/nature-research/editorial-policies/image-integrity">Digital Image Integrity Guidelines. and to the following points below:

-- that unprocessed scans are clearly labelled and match the gels and western blots presented in figures.
-- that control panels for gels and western blots are appropriately described as loading on sample processing controls
-- all images in the paper are checked for duplication of panels and for splicing of gel lanes.

Finally, please ensure that you retain unprocessed data and metadata files after publication, ideally archiving data in perpetuity, as these may be requested during the peer review and production process or after publication if any issues arise.

If there are additional or modified structures presented in the final revision, please submit the corresponding PDB validation reports.

Please note that all key data shown in the main figures as cropped gels or blots should be presented in uncropped form, with molecular weight markers. These data can be aggregated into a single supplementary figure item. While these data can be displayed in a relatively informal style, they must refer back to the relevant figures. These data should be submitted with the final revision, as source data, prior to acceptance, but you may want to start putting it together at this point.

SOURCE DATA: we urge authors to provide, in tabular form, the data underlying the graphical representations used in figures. This is to further increase transparency in data reporting, as detailed in this editorial (http://www.nature.com/nsmb/journal/v22/n10/full/nsmb.3110.html). Spreadsheets can be submitted in excel format. Only one (1) file per figure is permitted; thus, for multi-paneled figures, the source data for each panel should be clearly labeled in the Excel file; alternately the data can be provided as multiple, clearly labeled sheets in an Excel file. When submitting files, the title field should indicate which figure the source data pertains to. We encourage our authors to provide source data at the revision stage, so that they are part of the peer-review process.

Data availability: this journal strongly supports public availability of data. All data used in accepted papers should be available via a public data repository, or alternatively, as Supplementary Information. If data can only be shared on request, please explain why in your Data Availability Statement, and also in the correspondence with your editor. Please note that for some data types, deposition in a public repository is mandatory - more information on our data deposition policies and available repositories can be found below:
https://www.nature.com/nature-research/editorial-policies/reporting-standards#availability-of-data

We require deposition of coordinates (and, in the case of crystal structures, structure factors) into the Protein Data Bank with the designation of immediate release upon publication (HPUB). Electron microscopy-derived density maps and coordinate data must be deposited in EMDB and released upon publication. Deposition and immediate release of NMR chemical shift assignments are highly encouraged. Deposition of deep sequencing and microarray data is mandatory, and the datasets must be released prior to or upon publication. To avoid delays in publication, dataset accession numbers must be supplied with the final accepted manuscript and appropriate release dates must be indicated at the galley proof stage.

While we encourage the use of color in preparing figures, please note that this will incur a charge to partially defray the cost of printing. Information about color charges can be found at http://www.nature.com/nsmb/authors/submit/index.html#costs

Nature Structural & Molecular Biology is committed to improving transparency in authorship. As part of our efforts in this direction, we are now requesting that all authors identified as 'corresponding author' on published papers create and link their Open Researcher and Contributor Identifier (ORCID) with their account on the Manuscript Tracking System (MTS), prior to acceptance. This applies to primary research papers only. ORCID helps the scientific community achieve unambiguous attribution of all scholarly contributions. You can create and link your ORCID from the home page of the MTS by clicking on 'Modify my Springer Nature account'. For more information please visit please visit www.springernature.com/orcid.

Please use the link below to submit your revised manuscript and related files:

[READACTED]

Note: This URL links to your confidential home page and associated information about manuscripts you may have submitted, or that you are reviewing for us. If you wish to forward this email to co-authors, please delete the link to your homepage.

We look forward to seeing the revised manuscript and thank you for the opportunity to review your work.

Sincerely,

Carolina

Carolina Perdigoto, PhD

Chief Editor
Nature Structural & Molecular Biology
orcid.org/0000-0002-5783-7106

Referee expertise:

Referee #1: cryo-EM of transcription complexes

Referee #2: Pol I; structural biology

Reviewers' Comments:

Reviewer #1:
Remarks to the Author:
Here Misiaszek et al., present the high-resolution cryo-EM structures of human Pol I elongation, open, and RRN3 bound complexes. The structures yield a number of surprises, such as a single subunit stalk and a well-defined RNA hairpin in the RNA exit channel of the enzyme. These structures allow for the careful mapping of various disease mutations and for extensive phylogenetic and structural comparisons. This manuscript will be of broad interest to the transcription community and is a significant contribution to our understanding of multi-subunit RNA polymerases. However, there are a number of minor issues the authors should address prior to publication.

Specific comments:
Line 146 The authors are able to visualize a hairpin RNA in the RNA exit tunnel of human Pol I, which is an exciting observation. They comment that the RNA exit tunnel is wider in Pol I than other multi-subunit mammalian RNA polymerases. How wide are the RNA tunnels the human OC and RRN3 Pol I structures? There are suggestions that mammalian Pol II could also accommodate a hairpin RNA in the exit channel. It is thus important to indicate whether the observation of the widened RNA exit channel is specific to the nucleic acid sequence employed here or if this is a general feature of human Pol I.

Lines 177-178 The authors mention that the human Pol I stalk is “less tightly bound to the core compared to the yeast Pol I stalk”. The authors appear to make this suggestion based on interactions observed in previous crystal structures of yeast enzymes compared to the human enzyme. Such a bold statement should be supported by data, such as measured binding affinity. If the authors are unable to provide data supporting this statement, they should make the language in this sentence more suggestive.

Lines 220-221 RPA34 uses a proline rich extension to anchor itself to the Pol I core. Yeast A34.5 appears to function similarly, however, uses a different sequence to achieve this. It would help to have a structural overlay of the two extensions on Pol I to more easily visualize this difference (in addition to the provided sequence alignment).

Lines 391-392 Disease mutations associated with HLD and TCS are mapped to human Pol I. It is

stated that "HLD-associated mutations in this region cannot directly impair the activity of Pol I." Although this is likely the case, not being able to structurally resolve a region of protein does not absolve it from having a role in disease. The language here should be toned down to make this statement more suggestive.

Figure 1b- It would help if the percentage identity column was marked as a comparison between S. cerevisiae and human. Unclear in the figure what this means although it is stated in the legend.

Figure 1d, e- Rotations used for the cryo-EM maps and cartoon representations of the Pol I structure should be shown in the same orientations. Currently, the right panels in d and e are in different rotational states and thus cannot be easily compared.

Extended data Figure 1, Extended data Figure 5B- FSC curves for all deposited maps should be provided. Currently FSC curves for Map B, D and G are missing. Local resolution estimates for these maps should also be provided.

Extended data Figure 3C- The yeast and human structural comparison is good, however, it would help if the yeast (light pink) coloring was indicated in the structural overlay.

Figure 5 What residues do RRN3 S199, Thr 200 interact with on RPA43? How could phosphorylation disrupt this interaction? It is fine if the resolution of the structure does not allow for this identification, but if it should be stated in the legend somewhere. V156 and Q108- why are these residues marked? They are not mentioned in the figure legend or text and do not seem to be viable phosphorylation targets.

The authors mention that the active site Mg2+ is not visible in the open complex structure. This seems to suggest it is visible in the EC structure (also suggested by the inclusion of a Mg2+ in Table 1). The authors should thus display the active site Mg2+ in Extended Figure 6b.

Reviewer #2:
Remarks to the Author:
The manuscript by Misiaszek et al. reports cryoEM structures of human RNAP I in different functional states, i.e. elongation complex, bound to Rrn3 and bound to an open DNA. The structures reveal features that are new respect to reported structures of the cerevisiae and pombe counterparts: (i) a single-subunit stalk with increased flexibility, (ii) a large RNA exit channel that can harbor dsRNA, and (iii) minor features of the A49/34 heterodimer. In addition, mapping of disease-associated mutations on the structure provides structural basis of genetic disorders.

The data are overall technically sound and provide relevant information to illuminate specific regulatory aspects of human rDNA transcription, with implications in development, aging, response to stress and cancer. The manuscript is clearly written and figures are informative. Nevertheless, there are a few points that should be addressed by the authors.

My main concern refers to the assignment of the RNAP I structure in complex with a full bubble scaffold as a canonical elongation complex. The in vitro transcription assay shows poor RNA extension and cleavage activities over the designed scaffold (Fig 1c). For comparison, human RNAP III purified in

equivalent conditions showed significantly stronger activities (Ext Data Fig 1 in Girbig 2021, NSMB). Apart from the enzyme, the main difference is that in the current report the scaffold was prepared by addition of pre-heated RNA oligo onto ice-cold DNA bubble, while in Girbig 2021 the mix is heated to 45 degrees after RNA oligo addition. This may have caused self-association of the RNA oligo, as observed by the authors in the RNA exit channel (Fig 2a-b). The strong negative charge of the channel (Fig 2e, line 145) and the size of the contact surface suggest that the channel-dsRNA interaction is strong. Moreover, the structure is reminiscent of the paused elongation complex induced by an RNA hairpin reported for E.coli RNAP (Kang 2018, Mol Cell), which the authors reference but do not use for comparison. The dsRNA in both structures is 5 nt long and only 1 nt upstream of the hybrid. Overall, this suggests that the structure may represent a paused rather than a canonical elongation complex. To address this, the authors should both enlarge the analysis of their structure and perform additional primer extension assays. Structural analysis should include an exhaustive comparison with PDB 6ASX (Kang 2018), both at the dsRNA region and around the active site. The latter is important to determine the translocation state (pre, post or intermediate) and should include a figure with the map (preferably as mesh) and model around the active site. The authors should also compare with the yeast RNA exit channel to address if this is specific for human RNAP I. The transcription assay should be compared with a dsRNA-free scaffold (for example, a 9-mer RNA lacking the 5' extension) and include a control of yeast RNAP I on both scaffolds, i.e. with and without the 5' extension. Finally, the authors used equimolar amounts of the three oligos for scaffold preparation but the RNA oligo dimerizes, suggesting that a significant portion of DNA is devoid of RNA. Does this show up as a discrete population upon cryoEM data processing?

The phylogenetic analysis of stalk subunits and neighboring domains, based on structural data showing that the human RNAP I stalk only contains RPA43, is notable. However, the structural description of the stalk should be improved. First, how does the subdomain architecture of hRPA43 compare to its cerevisiae and pombe counterparts? A graph should be presented like in Ext Data Fig 3a. Second, the authors briefly mention that human RNAP I dimerization is not observed (line 280) but this statement is vague. Dimerization of cerevisiae (Pilsl 2016, Nat Comm) or pombe (Heiss 2021, Nat Comm) in solution has only been observed under special biochemical conditions. Have the authors explored conditions for dimer formation (like those in Pilsl 2016 or Heiss 2021) and observed no dimers? Third, the authors state that the stalk is less tightly bound to the core compared to its yeast counterpart (line 177). In Ext Data Fig 1, several particle groups lacking the stalk are discarded, which might account for 12-subunit RNAP I. Do these reconstructions lack the complete stalk or they exhibit density for the Tip (core anchoring) domain?

Both the RRN3-bound and open complex structures present a preferential view (Ext Data Fig 5c-d) that can produce elongated maps. The authors mention that they observe streaky density in the RRN3 complex (line 852). To evaluate the effect of this preferred orientation, the authors should report the 3D-FSC (Tan 2017, Nat Meth), with FSCs in the best and worse orientations, histogram distribution and sphericity.

Regarding the open complex structure, it seems that only the downstream DNA is observed. If that is the case, how much does this really resemble an open complex? At least weak density for the template strand or part of upstream DNA could be expected. The latter could be tested with focused 3D classification using appropriate masks.

Minor points:

Line 91. Include cites for Pilsl 2016, Nat Comm and Torreira 2017, eLife

Line 127. Nucleotide instead of 'nuclotide'

Line 133. The citation seems incorrect, as the EC structure in Tafur 2016, Mol Cell (PDB 5M5X) only has 5 nt in the exit channel

Line 134. In the text, the authors should make clear that dsRNA formation involves 2 RNA oligos and is not a hairpin

Line 184. Rpb4 is not the smaller stalk subunit in Pol II, as stated

Line 216. What do the authors mean by "perfect hydrophobic stretch"?

Lines 648 and 651. Report resin volume for strep-tactin and monoQ

Ext Data Fig 7. Include a panel with superposition of human RNAP I density and yeast DNA mimicking loop

Author Rebuttal to Initial comments

Point-by-point Response to the Reviewers

Reviewer #1:

Here Misiaszek et al., present the high-resolution cryo-EM structures of human Pol I elongation, open, and RRN3 bound complexes. The structures yield a number of surprises, such as a single subunit stalk and a well-defined RNA hairpin in the RNA exit channel of the enzyme. These structures allow for the careful mapping of various disease mutations and for extensive phylogenetic and structural comparisons. This manuscript will be of broad interest to the transcription community and is a significant contribution to our understanding of multi-subunit RNA polymerases. However, there are a number of minor issues the authors should address prior to publication.

Response: We are pleased that the Reviewer #1 acknowledges the broad interest of our manuscript to the transcription community. We address the different points raised by the referee in the following responses.

Specific comments:

Line 146 The authors are able to visualize a hairpin RNA in the RNA exit tunnel of human Pol I, which is an exciting observation. They comment that the RNA exit tunnel is wider in Pol I than other multi-subunit

mammalian RNA polymerases. How wide are the RNA tunnels the human OC and RRN3 Pol I structures? There are suggestions that mammalian Pol II could also accommodate a hairpin RNA in the exit channel. It is thus important to indicate whether the observation of the widened RNA exit channel is specific to the nucleic acid sequence employed here or if this is a general feature of human Pol I.

Response: As shown in Fig. 4 and stated in lines 269-270 the RPA49 tWH domain is disordered in both Pol I OC and Pol I RRN3 structures. Thus, the RNA exit tunnel remains open from one side in the Pol I OC and Pol I RRN3 structures, preventing us from measuring its width in the exact same location as reported in Fig. 2e. We have therefore measured the orthogonal width, which is 25Å for Pol I EC and 26Å for Pol I OC and Pol I – RRN3. This is only a small difference, which can be attributed to the structural rearrangements caused by cleft opening (shown in Fig. 4c). Thus, a wide RNA exit tunnel is a general feature of Pol I that allows accommodating RNA structures independent of their sequence. Another Pol I specific feature is the tWH domain that becomes ordered in the EC state. It shields the RNA exit tunnel and extends the positively charged patch as shown in Fig. 2e.

Lines 177-178 The authors mention that the human Pol I stalk is "less tightly bound to the core compared to the yeast Pol I stalk". The authors appear to make this suggestion based on interactions observed in previous crystal structures of yeast enzymes compared to the human enzyme. Such a bold statement should be supported by data, such as measured binding affinity. If the authors are unable to provide data supporting this statement, they should make the language in this sentence more suggestive.

Response: We acknowledge that our phrasing did not directly correlate with the presented data. We therefore changed it into the more appropriate statement comparing the size of the interaction interface between the stalk and core in human and yeast Pol I: *"in human Pol I the binding interface between the stalk and core is smaller compared to yeast Pol I".*

Lines 220-221 RPA34 uses a proline rich extension to anchor itself to the Pol I core. Yeast A34.5 appears to function similarly, however, uses a different sequence to achieve this. It would help to have a structural overlay of the two extensions on Pol I to more easily visualize this difference (in addition to the provided sequence alignment).

Response: We added panel d in Extended Data Fig. 3 showing the superposition of the yeast and human extensions.

Lines 391-392 Disease mutations associated with HLD and TCS are mapped to human Pol I. It is stated that "HLD-associated mutations in this region cannot directly impair the activity of Pol I." Although this is likely the case, not being able to structurally resolve a region of protein does not absolve it from having a role in disease. The language here should be toned down to make this statement more suggestive.

Response: We have toned down the statement by stating that the *"HLD-associated mutations in this region are less likely to directly impair the activity of Pol I"*.

Figure 1b- It would help if the percentage identity column was marked as a comparison between S. cerevisiae and human. Unclear in the figure what this means although it is stated in the legend.

Response: We changed the label in Fig. 1b into *"% Identity human vs yeast"*.

Figure 1d, e- Rotations used for the cryo-EM maps and cartoon representations of the Pol I structure should be shown in the same orientations. Currently, the right panels in d and e are in different rotational states and thus cannot be easily compared.

Response: We now show cryo-EM maps and cartoon representations in Fig. 1d and e in the same orientations.

Extended data Figure 1, Extended data Figure 5B- FSC curves for all deposited maps should be provided. Currently FSC curves for Map B, D and G are missing. Local resolution estimates for these maps should also be provided.

Response: We included the FSC curves and local resolution estimates as well as angular distribution maps for all structures. For Maps A and B this information is provided in Extended Data Fig. 1 and for Maps D-G in Extended Data Fig. 5.

Extended data Figure 3C- The yeast and human structural comparison is good, however, it would help if the yeast (light pink) coloring was indicated in the structural overlay.

Response: We assume that the reviewer was referring to the right panel in Extended Data Fig. 4c and to this panel we added the labels "human RPA49" and "yeast A49" in purple and pink, respectively.

Figure 5 What residues do RRN3 S199, Thr 200 interact with on RPA43? How could phosphorylation disrupt this interaction? It is fine if the resolution of the structure does not allow for this identification, but if it should be stated in the legend somewhere. V156 and Q108- why are these residues marked? They are not mentioned in the figure legend or text and do not seem to be viable phosphorylation targets.

Response: At the resolution we obtained in this part of the stalk, we are not able to confidently say which residues of RPA43 would interact with the phosphorylated residues of RRN3 and we have now included this statement in the legend of Fig. 5: "*The local resolution does not allow to unambiguously identify the contacting residues from RPA43*.". We speculate that the inactivating role of this modification could be due to charge repulsion. The stalk surface is negatively charged in this area (Fig. R1) and phosphorylation of S199 and T200 could lead to charge repulsion. We now included this speculation in text on page 12: "*We speculate that phosphorylation at these two positions, adjacent to each other, may function as a phospho-switch and disrupt the interaction between RRN3 and RPA43 for example by changing the charge distribution in the interaction area*.".

We agree that the labelling of Fig. 5a was confusing and thank the reviewer for pointing this out. Labels such as V156 and Q108 marked the boundaries of ordered regions. We now removed those labels and instead only label the N- and C-terminus of RRN3. We now also indicate the disordered regions more clearly as dashed lines.

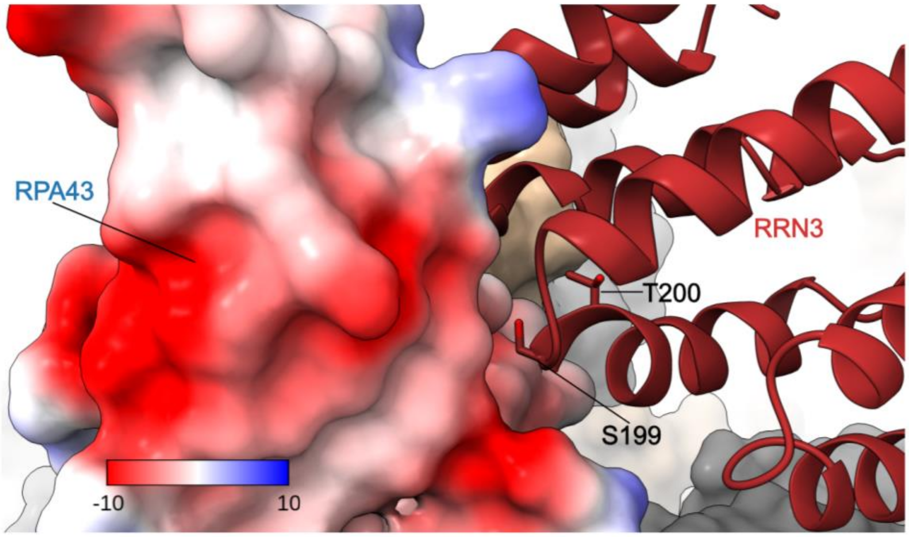

Fig. R1: Surface charge of the stalk at the RRN3 binding site. The stalk subunit RPA43 is shown in surface representation colored by electrostatic potential in kcal/(mol·e) at 298 K from negative (red) to positive (blue) calculated using ChimeraX. RRN3 is depicted in cartoon representation. Only residues S199 and T200 are shown in stick representation.

The authors mention that the active site Mg2+ is not visible in the open complex structure. This seems to suggest it is visible in the EC structure (also suggested by the inclusion of a Mg2+ in Table 1). The authors should thus display the active site Mg2+ in Extended Figure 6b.

Response: We have indeed chosen an unfortunate orientation for the display of the active site in the Extended Data Fig. 6b which occluded the Mg^{2+} ion. We have now changed the orientation such that the Mg^{2+} ion is visible and can be labeled.

__**Reviewer #2:**__

The manuscript by Misiaszek et al. reports cryoEM structures of human RNAP I in different functional states, i.e. elongation complex, bound to Rrn3 and bound to an open DNA. The structures reveal features that are new respect to reported structures of the cerevisiae and pombe counterparts: (i) a single-subunit stalk with increased flexibility, (ii) a large RNA exit channel that can harbor dsRNA, and (iii) minor

features of the A49/34 heterodimer. In addition, mapping of disease-associated mutations on the structure provides structural basis of genetic disorders.
The data are overall technically sound and provide relevant information to illuminate specific regulatory aspects of human rDNA transcription, with implications in development, aging, response to stress and cancer. The manuscript is clearly written and figures are informative. Nevertheless, there are a few points that should be addressed by the authors.

Response: We are pleased that the Reviewer #2 finds our study of scientific relevance and technically sound. We have addressed the specific points as detailed below.

My main concern refers to the assignment of the RNAP I structure in complex with a full bubble scaffold as a canonical elongation complex.

Response: We thank the reviewer for pointing out this possibility, which we have considered in detail guided by the reviewer's suggestions as detailed below. After performing the suggested analyses we are still convinced that the Pol I EC structure presents a canonical elongation complex in post-translocated state, which is expected given that no NTPs were added to the cryo-EM sample preparation. Below we include our answers in detail to the specific points raised by this reviewer.

In summary, we have introduced the following changes to the manuscript to address the reviewer's concerns: (i) We replaced the transcription assay in Fig. 1c with a new one showing more resolved bands which we have run alongside human RNA polymerase III for comparison (Figure R2); (ii) In the text we now specify that the Pol I EC structure shows a post-translocated state and we included a new panel e in Fig. 2 showing the human Pol I active site; (iii) in Fig. 2 panels c and d we now indicate the conservation between human and yeast Pol I residues making contacts with the RNA; (iv) we added in Fig. 2f an additional panel showing the charge distribution of the RNA exit tunnel in yeast Pol I together with its width measurement and we specified in the text that the wider RNA exit tunnel is likely a conserved, Pol I specific feature.

The in vitro transcription assay shows poor RNA extension and cleavage activities over the designed scaffold (Fig 1c). For comparison, human RNAP III purified in equivalent conditions showed significantly stronger activities (Ext Data Fig 1 in Girbig 2021, NSMB). Apart from the enzyme, the main

difference is that in the current report the scaffold was prepared by addition of pre-heated RNA oligo onto ice-cold DNA bubble, while in Girbig 2021 the mix is heated to 45 degrees after RNA oligo addition. This may have caused self-association of the RNA oligo, as observed by the authors in the RNA exit channel (Fig 2a-b).

Response: To address this comment of the referee we prepared a transcription assay where human Pol I and Pol III were treated in exactly the same manner and were tested on a scaffold annealed under the same conditions (addition of pre-heated RNA oligo onto ice-cold DNA bubble). As shown in Fig. R2, no significant differences in activity could be observed, especially when taking into account the disappearance of the primer upon addition of the nucleotides. This shows that human Pol I and Pol III are comparable in their *in vitro* activities. While the side-to-side comparison of the transcriptional activities of human Pol I and Pol III directly addresses the concern of reviewer #2, we don't think the side-to-side comparison adds new important information to the manuscript. Therefore, the results shown in Fig. R2 have not been included into the revised version of the manuscript. We have however included the panel showing human Pol I transcription activity in Fig. 1c as the individual bands here are more resolved compared to the previously displayed transcription assay.

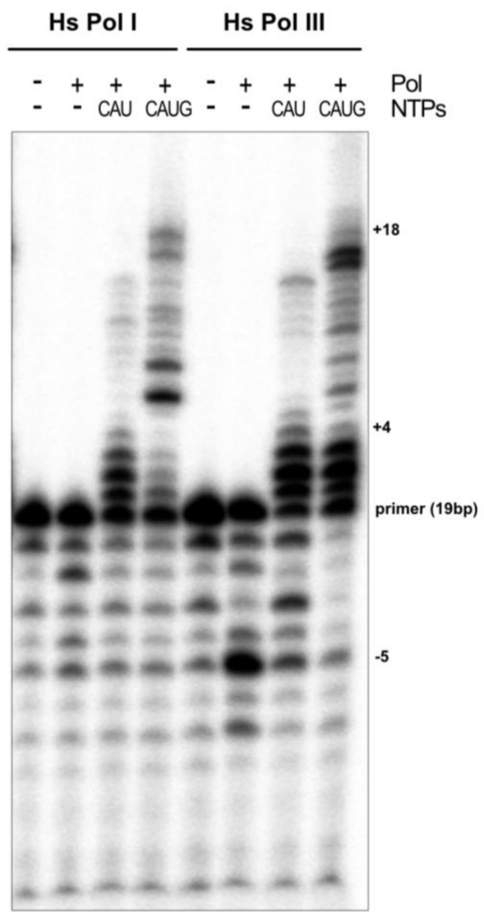

Fig. R2: Comparison of the transcription activity of human Pol I and Pol III. A 19-bp RNA primer radioactively labelled on the 3' end was used. Presence or absence of the respective RNA polymerase and nucleotides is indicated on the top.

The strong negative charge of the channel (Fig. 2e, line 145) and the size of the contact surface suggest that the channel-dsRNA interaction is strong. Moreover, the structure is reminiscent of the paused elongation complex induced by an RNA hairpin reported for E.coli RNAP (Kang 2018, Mol Cell), which the authors reference but do not use for comparison. The dsRNA in both structures is 5 nt long and only 1 nt upstream of the hybrid. Overall, this suggests that the structure may represent a paused rather than a canonical elongation complex.

To address this, the authors should both enlarge the analysis of their structure and perform additional primer extension assays. Structural analysis should include an exhaustive comparison with PDB 6ASX (Kang 2018), both at the dsRNA region and around the active site. The latter is important to determine the translocation state (pre, post or intermediate) and should include a figure with the map (preferably as mesh) and model around the active site. The authors should also compare with the yeast RNA exit channel to address if this is specific for human RNAP I.

The transcription assay should be compared with a dsRNA-free scaffold (for example, a 9-mer RNA lacking the 5' extension) and include a control of yeast RNAP I on both scaffolds, i.e. with and without the 5' extension.

Response: We thank reviewer #2 for these suggestions as they prompted us to analyze the active site, nucleic acid contacts and RNA exit channel in human and yeast Pol I as well as in the bacterial RNAP more thoroughly.

First, we looked in detail at the active site and the RNA exit tunnel in human Pol I, yeast Pol I in the elongating state and bacterial RNAP in the paused state (Fig. R3a). When inspecting the active site (Fig. R3a, top panel), we noted that in the human Pol I structure, as well as in the yeast Pol I EC structure, the free template base at the i+1 position is clearly visible, which characterizes the canonical elongating, post-translocated state. In contrast, in the bacterial RNAP there is no free template base and the DNA-RNA hybrid is titled as expected from the paused state. The top panel, left showing the active site of human Pol I has been added as Fig. 2e accompanied by the statement in the manuscript, page 5 marking the human Pol I EC structure as post-translocated state: "*So far, RNA secondary structure elements in the exit tunnel of RNA polymerases have only been captured in bacterial RNA polymerase in a paused state*[41]. *Human Pol I EC structure represents a post-translocated state with an empty i+1 site allowing accommodation of the incoming nucleotide (Fig. 2e).*"

Further, the structure of the RNA exit tunnel is very similar, both at it most narrow portion (Fig. R3a, middle panel) as well as at the widest point (Fig. R3a, bottom panel) in human and yeast Pol I (now depicted in Fig. 2f) and in bacterial RNAP. The general structural elements present are conserved between human Pol I and bacterial RNAP with the β' subunit occupying the space taken up by RPA49 in human Pol I to close the RNA exit tunnel. This similar architecture constricts the possible conformations

of the RNA leading to very similar RNA structures in the exit tunnel despite different functional states of the complex. Additionally, in the bacterial RNAP, pausing is induced by the swivel motion of the upper clamp (Fig. R3b, left panel). This movement is most clearly visible in the displacement of the clamp helices between the EC and paused state of bacterial RNAP, while such displacement is not observed between the yeast and human Pol I (Fig. R3b, right panel). Thus, we are convinced that even though the RNA structure is similar between human Pol I and bacterial RNAP, the functional state of the two complexes is different with human Pol I representing the canonical elongation post-translational state.

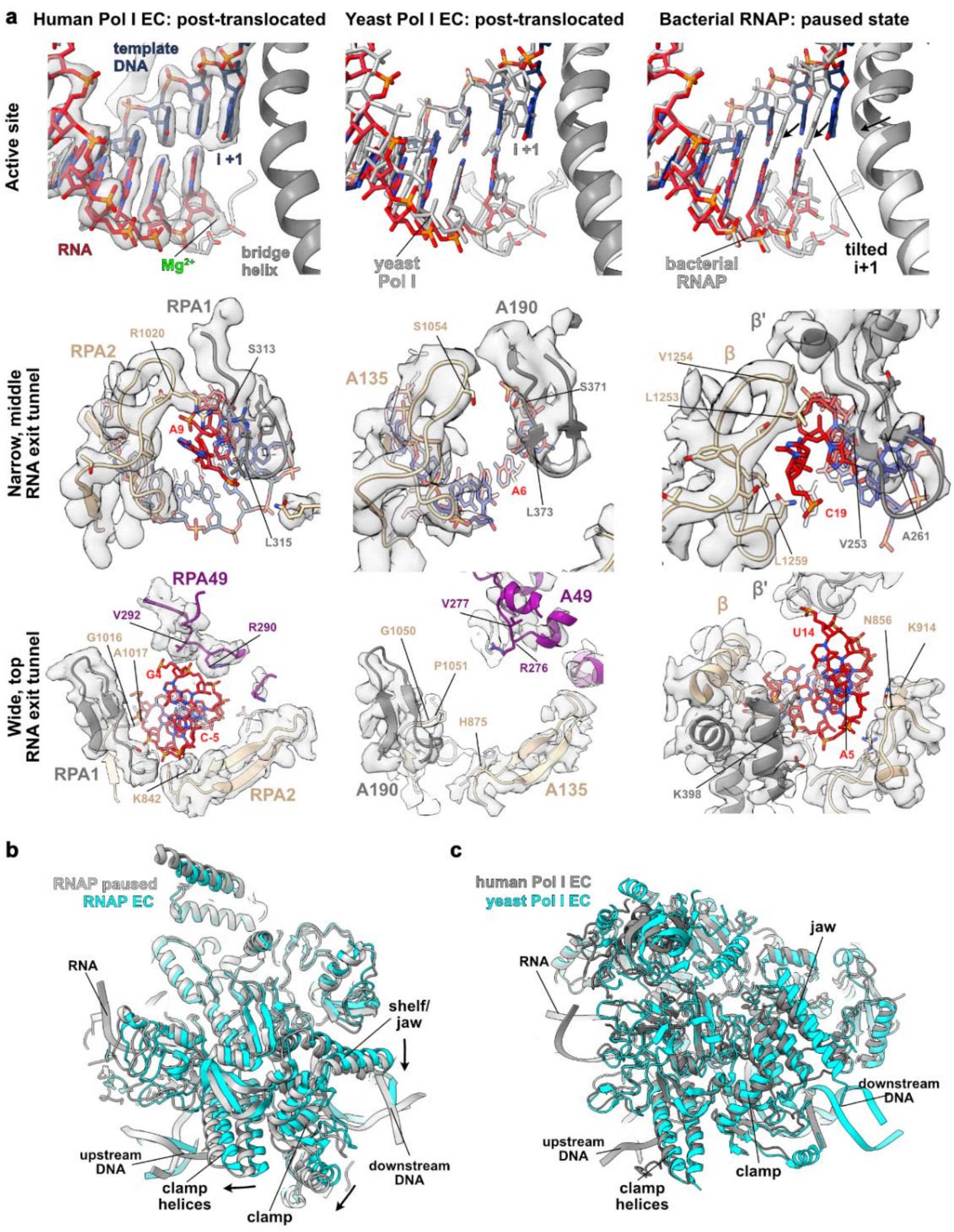

Fig. R3: Comparison of the functional state of human Pol I, yeast Pol I and bacterial RNAP. a Nucleic acid templates and their surrounding at the active site (top lane), in the most narrow point in the RNA exit tunnel (middle lane) and at the widest point in the RNA exit tunnel (bottom lane) between human Pol I EC (left panel), yeast Pol I (PDB: 5M64) (middle panel) and bacterial RNAP (PDB: 6ASX) (right panel). In the human Pol I active site, the cryo-EM density for the nucleic acids is shown in grey.

Yeast Pol I and bacterial RNAP (silver) are superimposed onto human Pol I (dark grey, nucleotides in color). The shift of the bridge helix and DNA bases in the bacterial RNAP in comparison to human Pol I is indicated with black arrows. In the panels showing the RNA exit tunnel, cryo-EM density corresponding to the structural elements forming the tunnel is shown in grey. Residues within 5 Å from the RNA are shown in stick representation while residues making contacts to the RNA are annotated. In the yeast Pol I structure, residues homologous to the ones marked in human Pol I are also annotated. **b** Superimposition of the clamp regions between (left) bacterial RNAPs in (silver) paused state (PDB: 6ASX) and (cyan) elongating state (PDB: 6ALF) and (right) Pol I from (silver) humans and (cyan) yeast in elongating state (PDB: 5M64). Main structural elements are annotated and the black arrows on the left panel indicate displacement induced by the swivel motion of the upper clamp in transition from elongating to paused complex in bacterial RNAP.

We analyzed the conservation of yeast and human Pol I residues contacting the nucleic acid scaffold. Identical, conserved and non-conserved human and yeast Pol I residues contacting the nucleic acid scaffold are depicted in Fig. R4 showing that most residues are identical between the two species. Conservation of yeast and human Pol I residues making contacts to the RNA in the exit tunnel is now depicted in Figs. 2c, d of the revised manuscript.

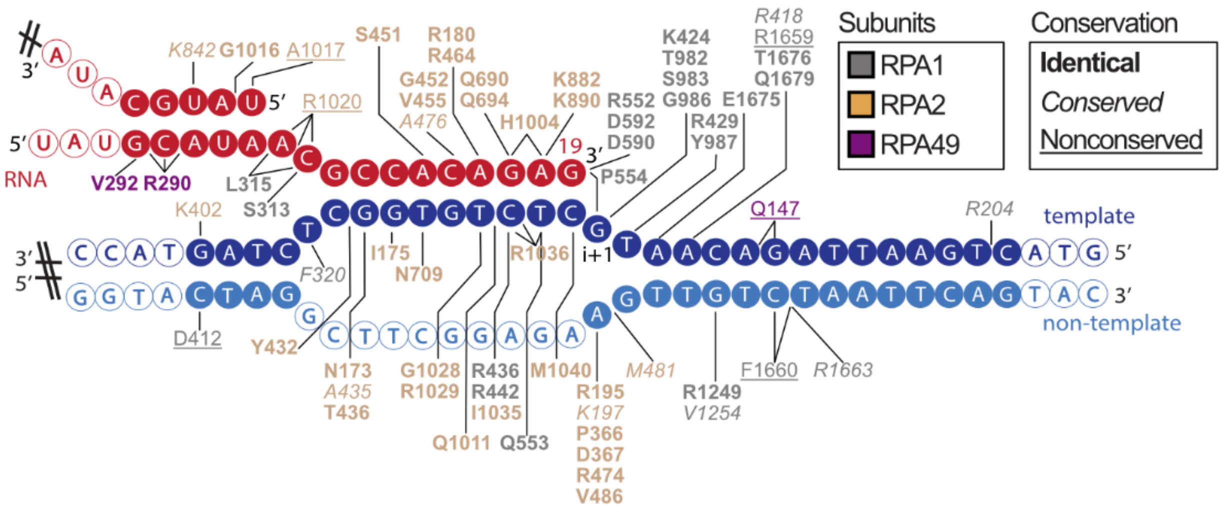

Fig. R4: Residues contacting the nucleic acid scaffold in human Pol I. The nucleic acid scaffold is presented in cartoon representation with colored circles marking nucleotides visible in the cryo-EM density and open circles marking nucleotides lacking cryo-EM density and that have not been included

into the final model. Human Pol I residues making contacts with nucleotides within 4 Å radius are depicted.

Finally, we compared the transcriptional activity of human and yeast Pol I on scaffolds with RNA that was self-complementary and with RNA that was single-stranded (Fig. R5). We did not note any significant differences between human and yeast Pol I, however the activity seemed to be slightly higher when the double-stranded RNA primer was used. This observation would support the hypothesis suggested by Turowski et al., Mol Cell, 2020 that the formation of RNA structures may promote faster transcription elongation.

To summarize, we think that the general features of the RNA exit tunnel are conserved between human and yeast Pol I. We have thus adjusted the text in the "Double-stranded RNA in the exit tunnel" section, page 5 to account for this observation. In contrast, the comparison between the human and yeast Pol I using an RNA primer that dimerizes and a single-stranded RNA primer depicted in Fig. R5 requested as additional control by reviewer #2 has not been included into the revised manuscript.

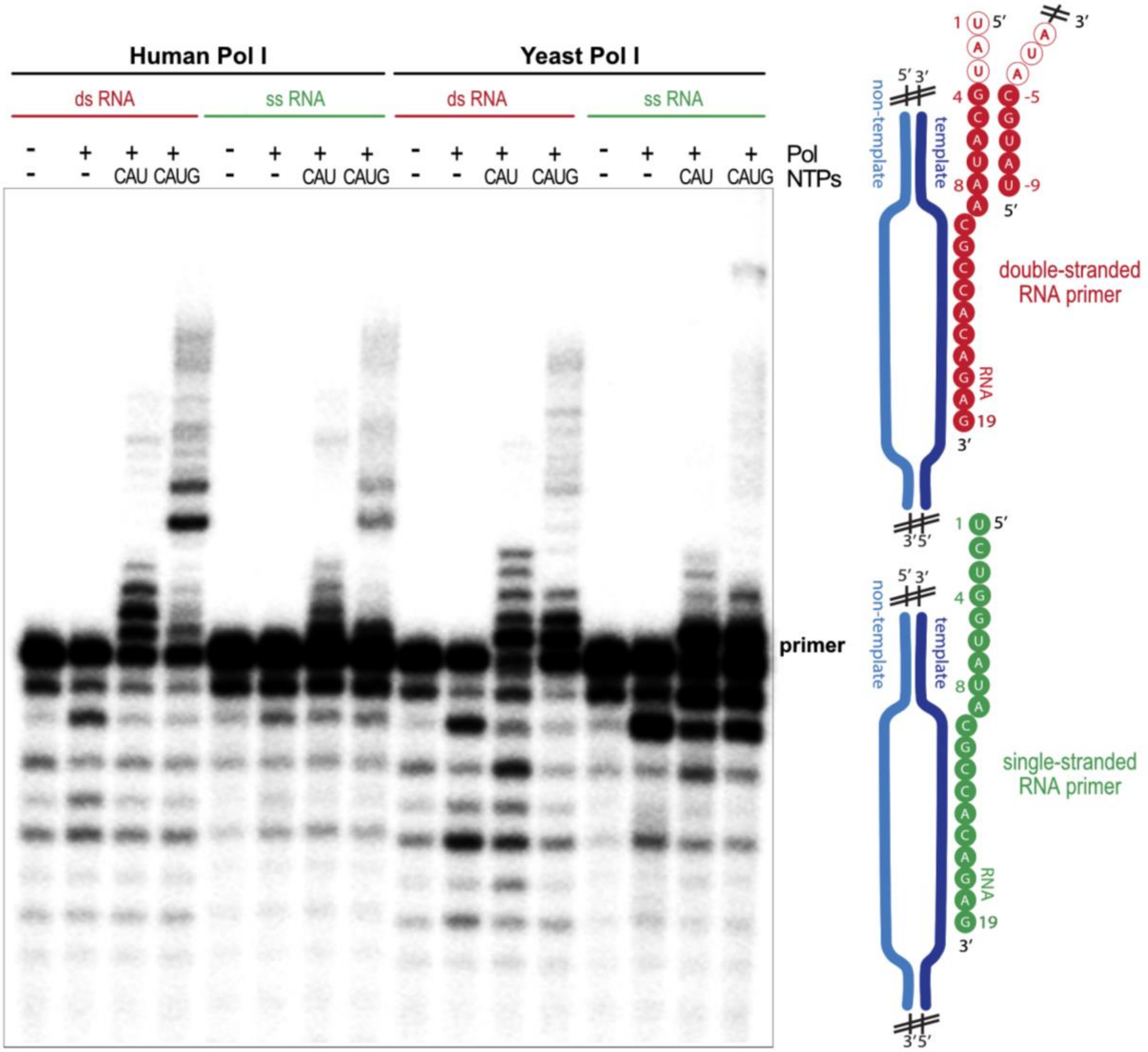

Fig. R5: Comparison of the transcriptional activity between human and yeast Pol I using an RNA primer that dimerizes and a single-stranded RNA primer. Used nucleic acid scaffolds in the cartoon representation are shown on the right. The double-stranded RNA primer has been used throughout the study and the single stranded primer has been designed to have similar base composition. Both primers were radioactively labelled on the 3’ end and annealed to the DNA scaffold as described in the Methods. Presence or absence of Pol I in the assay as well as addition of nucleotides is indicated on top.

Finally, the authors used equimolar amounts of the three oligos for scaffold preparation but the RNA oligo dimerizes, suggesting that a significant portion of DNA is devoid of RNA. Does this show up as a discrete population upon cryoEM data processing?

Response: In our analysis (as it is often the case in single particle analysis), only a small subset of particles were used for the final reconstruction. For Map A, 26% of the cleaned particles (199k out of 770k particles) were used, while to obtain Map B, 5% of the particles were used (37k out of 770k particles) – seen in Extended Data Fig. 1. We cannot exclude that in the discarded particles there is a sub-population of particles with a DNA scaffold devoid of RNA. Yet no such discrete population could be identified at any point. Additionally, we used an x1.5 molar excess of the DNA-RNA scaffold over Pol I. The transcription bubble that contains both, DNA and RNA, is more stable, leading to higher resolution structures which we selected for. Likely, the structure of the Pol I OC is reminiscent of how such a sub-population would look like.

The phylogenetic analysis of stalk subunits and neighboring domains, based on structural data showing that the human RNAP I stalk only contains RPA43, is notable. However, the structural description of the stalk should be improved. First, how does the subdomain architecture of hRPA43 compare to its cerevisiae and pombe counterparts? A graph should be presented like in Ext Data Fig 3a.

Response: In Fig. 3a we have now included a bar diagram comparison of human RPA43 subunit and its homologs in *S. cerevisiae* and *S. pombe*. We have also added a panel Fig. 3b with a structure superposition of human and *S. cerevisiae* subunits. We now refer to the new panels in the text at page 6: *"The A43 stalk subunit has similar domain architecture between yeast and humans…"*.

Second, the authors briefly mention that human RNAP I dimerization is not observed (line 280) but this statement is vague. Dimerization of cerevisiae (Pilsl 2016, Nat Comm) or pombe (Heiss 2021, Nat Comm) in solution has only been observed under special biochemical conditions. Have the authors explored conditions for dimer formation (like those in Pilsl 2016 or Heiss 2021) and observed no dimers?

Response: Due to limited amount of starting material we have not explored human Pol I dimerization under special biochemical conditions. Pol I dimerization has been extensively studied in *S. cerevisiae* and the regions within A43 subunit described as driving dimerization include residues 252-263 (Kostrewa *et*

al., Acta Crystallographica, 2015), 307-326 (Torreira *et al.*, eLife, 2017) and 263-315 (Engel *et al.*, Nature, 2013). Thus, we checked sequence similarity in those regions between *S. cerevisiae* and human (Fig. R6a). Interestingly, these regions show low sequence similarity making it unlikely that human Pol I would form dimers using the same structural elements. Furthermore, both Pilsl *et al.*, Nature, 2016 and Heiss *et al.*, Nat Comm, 2021 observed a significant fraction of particles forming dimers after unsupervised 2D classification of their cryo-EM samples. We have performed such unsupervised 2D classification and have not observed any dimers (Fig. R6b). Based on our current observations we therefore think that human Pol I does not undergo dimerization in a manner similar to yeast. In the revised version of our manuscript we mention this observation in the Methods section on page 25: "*No dimers were observed in 2D class averages.*".

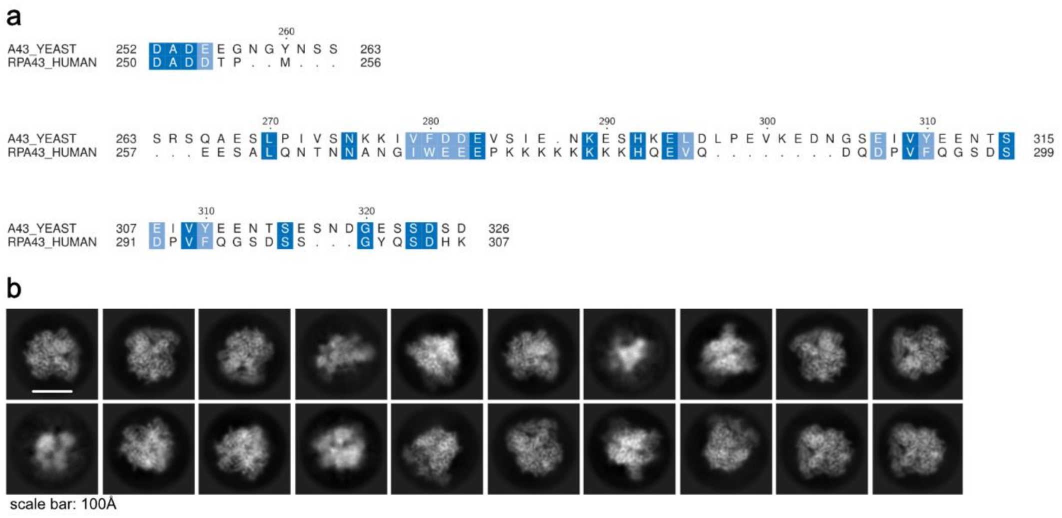

Fig. R6: Human Pol I dimerization is not observed. a Sequence alignment of the regions of yeast A43 subunit required for Pol I dimerization as noted (from top to bottom) by Kostrewa et al., Acta Crystallogr 2015; Torreira et al., eLife, 2017 and Engel et al., Nature, 2013. **b** Example classes from unsupervised 2D classification of the Pol I EC dataset. Scale bar: 100 Å.

Third, the authors state that the stalk is less tightly bound to the core compared to its yeast counterpart (line 177).

Response: We rephrased the statement about the stalk being less tightly bound to the core in human Pol I compared to the yeast Pol I. Instead, we now state on page 7 that the *"binding interface between the stalk and the core is smaller"* in human Pol I compared to yeast Pol I (see also our response to reviewer #1).

In Ext Data Fig 1, several particle groups lacking the stalk are discarded, which might account for 12-subunit RNAP I. Do these reconstructions lack the complete stalk or they exhibit density for the Tip (core anchoring) domain?

Response: The discarded particles have overall lower resolution, particularly in the region of upper clamp and stalk (Fig. R7a vs b). Thus, they do not contribute to the high-resolution structure. The density for the upper clamp in this class is of low quality (Fig. R7c). Yet, the cryo-EM density for the lower, core-anchoring domain of the stalk is clearly visible (Fig. R7d). In particular, density for RPA43 helix (residues 67-78) is visible (Fig. R7d) and is of similar quality as the elements visible within the clamp region in this class. Thus, while the cryo-EM density in the stalk region is of very poor quality, they still exhibit density for the core-anchoring domain of the stalk, and there is no indication that we observe a 12-subunit Pol I lacking the stalk in our dataset. We added a statement in the Methods section, page 26: *"In the discarded class all subunits were present with at least partial density, but the map quality was overall lower, especially in the upper clamp and stalk regions."*.

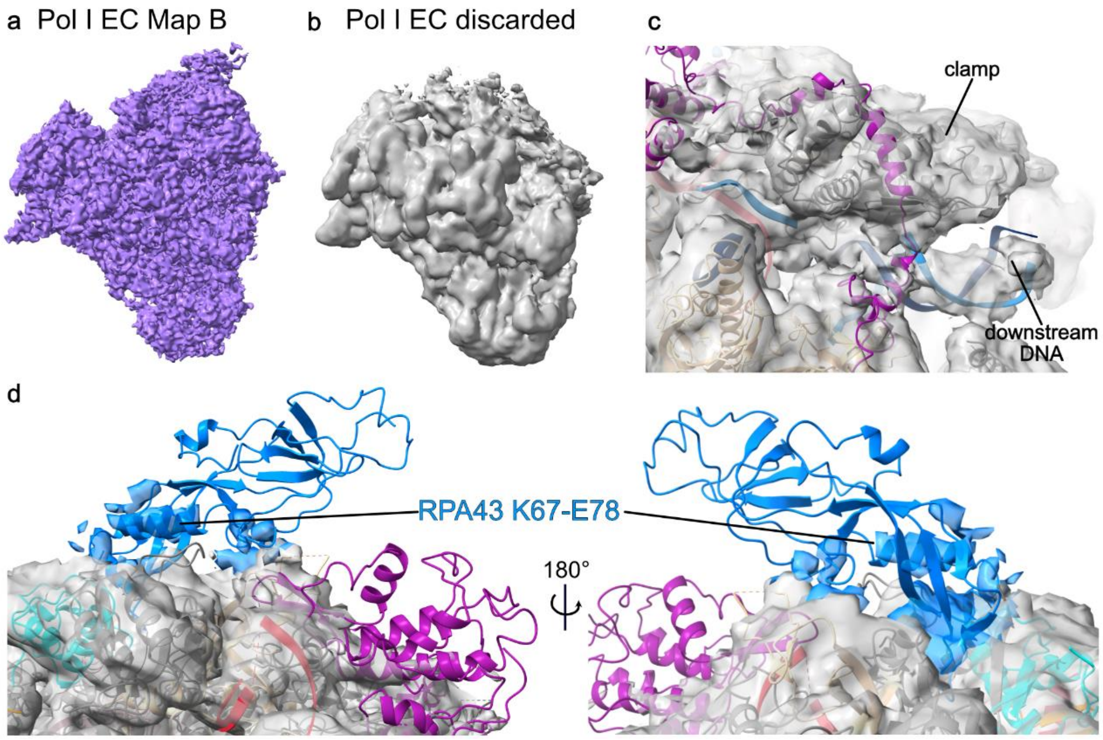

Fig. R7: Discarded particles contain the core-anchoring domain of the stalk. a Overall map quality of the selected class which gave rise to Map B. **b** Overall map quality of the discarded class. **c, d** Cryo-EM density from the discarded class is shown in grey. Pol I EC structure is shown in cartoon representation. **c** Zoom in on the clamp region. **d** Zoom in on the stalk region. The density corresponding to the RPA43 subunit is colored marine.

Both the RRN3-bound and open complex structures present a preferential view (Ext Data Fig 5c-d) that can produce elongated maps. The authors mention that they observe streaky density in the RRN3 complex (line 852). To evaluate the effect of this preferred orientation, the authors should report the 3D-FSC (Tan 2017, Nat Meth), with FSCs in the best and worse orientations, histogram distribution and sphericity.

Response: As suggested by reviewer #2, we performed the analysis using 3D-FSC (Fig. R8). In the text, we now report the values of sphericity for the Pol I-RRN3 maps (page 9 "*Focused refinement also alleviated effects of directional resolution anisotropy, improving sphericity from 0.796 to 0.836 as assessed by the 3D-FSC program*[54]."). However due to space limitations, we did not include Fig. R8 as an

Extended Data Figure even more as the observed directional resolution anisotropy is not very severe and we were still able to trace the pitch of the helices in our maps.

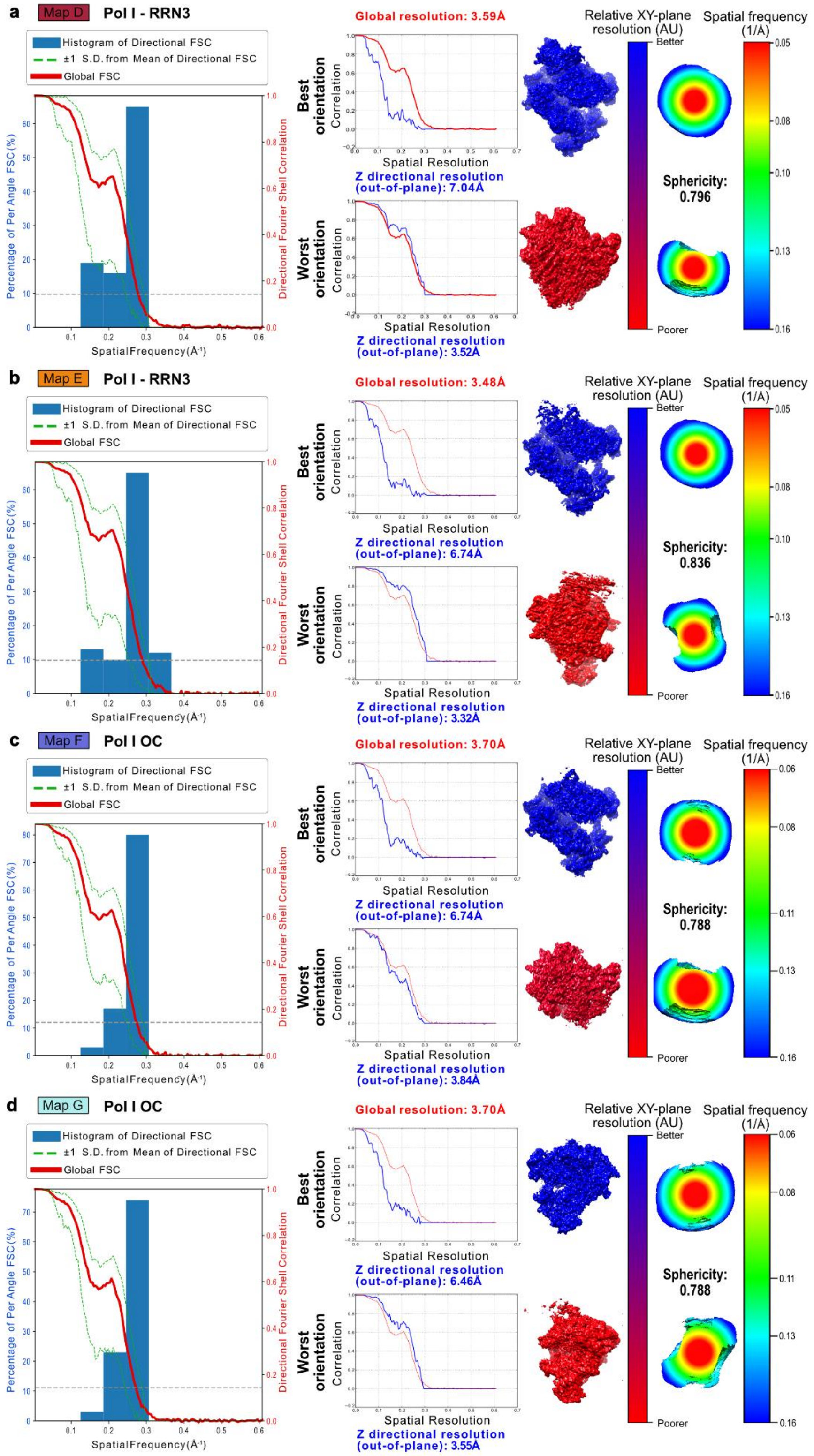
a
Map D
Pol I - RRN3
Histogram of Directional FSC
±1 S.D. from Mean of Directional FSC
Global FSC
Percentage of Per Angle FSC(%)
Directional Fourier Shell Correlation
SpatialFrequency(Å⁻¹)
Global resolution: 3.59Å
Best orientation
Correlation
Spatial Resolution
Z directional resolution (out-of-plane): 7.04Å
Worst orientation
Z directional resolution (out-of-plane): 3.52Å
Relative XY-plane resolution (AU)
Better
Poorer
Spatial frequency (1/Å)
Sphericity: 0.796
b
Map E
Pol I - RRN3
Global resolution: 3.48Å
Z directional resolution (out-of-plane): 6.74Å
Z directional resolution (out-of-plane): 3.32Å
Sphericity: 0.836
c
Map F
Pol I OC
Global resolution: 3.70Å
Z directional resolution (out-of-plane): 6.74Å
Z directional resolution (out-of-plane): 3.84Å
Sphericity: 0.788
d
Map G
Pol I OC
Global resolution: 3.70Å
Z directional resolution (out-of-plane): 6.46Å
Z directional resolution (out-of-plane): 3.55Å
Sphericity: 0.788

Fig. R8: 3D-FSC analysis of the Pol I – RRN3 and Pol I OC maps. a Map D, Pol I – RRN3. **b** Map E, Pol I – RRN3 obtained using focused refinement. **c** Map F, Pol I OC**. d** Map G Pol I OC obtained using focused classification of the nucleic acid scaffold. **a-d left to right** Histogram distribution of the directional FSC together with global FSC curve. FSC in the best (top) and worst (bottom) XY orientation. Sphericity measure together with the 3D FSC representation at best (top) and worst (bottom) orientation.

Regarding the open complex structure, it seems that only the downstream DNA is observed. If that is the case, how much does this really resemble an open complex? At least weak density for the template strand or part of upstream DNA could be expected. The latter could be tested with focused 3D classification using appropriate masks.

Response: We attempted to obtain a map with improved density for the nucleic acid scaffold using masked classification reported as Map G in Extended Data Fig. 5. We do observe density in the cleft, it is however weak and discontinuous which prevented us from confidently building a model for the unwound (and the upstream) part of the DNA scaffold. We have now included a statement at page 10 of our manuscript clarifying this observation: *"We could trace the double-stranded downstream DNA, but the fragmented cryo-EM density lining the cleft did not allow us to build confidently the unwound portion of the DNA indicating that it is not stably associated with the active site."*

Minor points:

Line 91. Include cites for Pilsl 2016, Nat Comm and Torreira 2017, eLife

Response: References for Pilsl *et al*., Nat Comm, 2016 and Torreira *et al*., eLife, 2017 have been added.

Line 127. Nucleotide instead of 'nuclotide'

Response: Typing error has been corrected.

Line 133. The citation seems incorrect, as the EC structure in Tafur 2016, Mol Cell (PDB 5M5X) only has 5 nt in the exit channel

Response: We were counting the length of the entire RNA primer, including the DNA-RNA hybrid. We have now made this sentence more explicit stating that: "*yeast Pol I between 7 and 13 nucleotides*

could be traced from the active site extending into the RNA exit tunnel[30]".

Line 134. In the text, the authors should make clear that dsRNA formation involves 2 RNA oligos and is not a hairpin

Response: This is now spelled out explicitly on page 5: *"The 5' end of the RNA oligonucleotide used is self-complementary to the 3' end of the exiting RNA oligonucleotide (Fig. 2b), and thus we placed an ideal double-stranded RNA helix (A-form) formed by base-pairing of two, separate RNA oligonucleotides into the exit tunnel that fitted well into the cryo-EM density and remained intact after refinement (Fig. 2a)."*

Line 184. Rpb4 is not the smaller stalk subunit in Pol II, as stated

Response: We changed the sentence referring to Rpb4 and Rpb7 to explicitly state that we mean the different sizes in the yeast Pol I: *"The stalk of yeast Pol I consists of two subunits: the larger yeast Pol I subunit A43, homologous to Rpb7 in Pol II and C25 in Pol III; and the smaller yeast Pol I subunit A14, homologous to Rpb4 in Pol II and C17 in Pol III[18,19]."*.

Line 216. What do the authors mean by "perfect hydrophobic stretch"?

Response: We replaced "perfect" by "continuous".

Lines 648 and 651. Report resin volume for strep-tactin and monoQ

Response: Added the resin volumes for the Strep-Tactin and the MiniQ 4.6 columns.

Ext Data Fig 7. Include a panel with superposition of human RNAP I density and yeast DNA mimicking loop.

Response: We have added two additional panels (right) with the yeast DNA mimicking loop into the Extended Data Fig. 7c, d.

Decision Letter, first revision:

21st Sep 2021

Dear Dr. Muller,

Thank you for submitting your revised manuscript "Cryo-EM structures of human RNA polymerase I" (NSMB-A45020A). It has now been seen by the original referees and their comments are below. The reviewers find that the paper has improved in revision, and therefore we'll be happy in principle to publish it in Nature Structural & Molecular Biology, pending minor revisions to satisfy the referees' final requests and include the suggested figures in the manuscript, and to comply with our editorial and formatting guidelines.

We are now performing detailed checks on your paper and will send you a checklist detailing our editorial and formatting requirements in about a week. Please do not upload the final materials and make any revisions until you receive this additional information from us.

Thank you again for your interest in Nature Structural & Molecular Biology Please do not hesitate to contact me if you have any questions.

Sincerely,

Carolina

Carolina Perdigoto, PhD
Chief Editor
Nature Structural & Molecular Biology
orcid.org/0000-0002-5783-7106

Reviewer #1 (Remarks to the Author):

The authors have addressed all reviewer concerns.

Reviewer #2 (Remarks to the Author):

The authors have convincingly addressed my previous concerns. In particular, they established that: (i) their EC structure is in the post-translocated state, (ii) the enlarged RNA exit tunnel is a conserved Pol I specific feature, and (iii) the stalk is tightly bound and unlikely to mediate Pol I dimerization. I would only point out that reviewer figures R4 and R5 are informative and the authors should consider including them into the supplementary material. In particular, Fig R5 suggests that dsRNA in the exit tunnel promotes elongation, thus supporting their interpretation of structural data (line 154).

Author Rebuttal, first revision:

Point-by-point Response to the Reviewers

Reviewer #1:

Remarks to the Author:

The authors have addressed all reviewer concerns.

Reviewer #2:

Remarks to the Author:

The authors have convincingly addressed my previous concerns. In particular, they established that: (i) their EC structure is in the post-translocated state, (ii) the enlarged RNA exit tunnel is a conserved Pol I specific feature, and (iii) the stalk is tightly bound and unlikely to mediate Pol I dimerization. I would only point out that reviewer figures R4 and R5 are informative and the authors should consider including them into the supplementary material. In particular, Fig R5 suggests that dsRNA in the exit tunnel promotes elongation, thus supporting their interpretation of structural data (line 154).

Response: Following the suggestion of Reviewer #2, we have now included Reviewer Figures R4 and R5 as Extended Data Fig.2a and b.

Final Decision Letter:

27th Oct 2021

Dear Dr. Müller,

We are now happy to accept your revised paper "Cryo-EM structures of human RNA polymerase I" for publication as a Article in Nature Structural & Molecular Biology.

Acceptance is conditional on the manuscript's not being published elsewhere and on there being no announcement of this work to the newspapers, magazines, radio or television until the publication date in Nature Structural & Molecular Biology.

Before the manuscript is sent to the printers, we shall make any detailed changes in the text that may be necessary either to make it conform with house style or to make it intelligible to a wider readership. If the changes are extensive, we will ask for your approval before the manuscript is laid out for production. Once your manuscript is typeset you will receive a link to your electronic proof via

email within 20 working days, with a request to make any corrections within 48 hours. Please read proofs with great care to make sure that the sense has not been altered. If you have queries at any point during the production process then please contact the production team at rjsproduction@springernature.com. Once your paper has been scheduled for online publication, the Nature press office will be in touch to confirm the details.

Please note that due to tight production schedules, proofs should be returned as quickly as possible to avoid delaying publication. If you anticipate any limitations to your availability over the next 2-4 weeks (such as vacation or traveling to conferences, etc.), please e-mail rjsproduction@springernature.com as soon as possible. Please provide specific dates that you will be unavailable and provide detailed contact information for an alternate corresponding author if necessary.

To assist our authors in disseminating their research to the broader community, our SharedIt initiative provides all co-authors with the ability to generate a unique shareable link that will allow anyone (with or without a subscription) to read the published article. Recipients of the link with a subscription will also be able to download and print the PDF.

As soon as your article is published, you can generate your shareable link by entering the DOI of your article here: <a href="http://authors.springernature.com/share">http://authors.springernature.com/share<a>. Corresponding authors will also receive an automated email with the shareable link

Note the policy of the journal on data deposition: http://www.nature.com/authors/policies/availability.html.

Your paper will be published online soon after we receive proof corrections and will appear in print in the next available issue. You can find out your date of online publication by contacting the production team shortly after sending your proof corrections. Content is published online weekly on Mondays and Thursdays, and the embargo is set at 16:00 London time (GMT)/11:00 am US Eastern time (EST) on the day of publication. Now is the time to inform your Public Relations or Press Office about your paper, as they might be interested in promoting its publication. This will allow them time to prepare an accurate and satisfactory press release. Include your manuscript tracking number (NSMB-A45020B) and our journal name, which they will need when they contact our press office.

About one week before your paper is published online, we shall be distributing a press release to news organizations worldwide, which may very well include details of your work. We are happy for your institution or funding agency to prepare its own press release, but it must mention the embargo date and Nature Structural & Molecular Biology. If you or your Press Office have any enquiries in the meantime, please contact press@nature.com.

You can now use a single sign-on for all your accounts, view the status of all your manuscript submissions and reviews, access usage statistics for your published articles and download a record of your refereeing activity for the Nature journals.

If you have not already done so, we strongly recommend that you upload the step-by-step protocols used in this manuscript to the Protocol Exchange. Protocol Exchange is an open online resource that

allows researchers to share their detailed experimental know-how. All uploaded protocols are made freely available, assigned DOIs for ease of citation and fully searchable through nature.com. Protocols can be linked to any publications in which they are used and will be linked to from your article. You can also establish a dedicated page to collect all your lab Protocols. By uploading your Protocols to Protocol Exchange, you are enabling researchers to more readily reproduce or adapt the methodology you use, as well as increasing the visibility of your protocols and papers. Upload your Protocols at www.nature.com/protocolexchange/. Further information can be found at www.nature.com/protocolexchange/about.

An online order form for reprints of your paper is available at https://www.nature.com/reprints/author-reprints.html. Please let your coauthors and your institutions' public affairs office know that they are also welcome to order reprints by this method.

Please note that <i>Nature Structural & Molecular Biology</i> is a Transformative Journal (TJ). Authors may publish their research with us through the traditional subscription access route or make their paper immediately open access through payment of an article-processing charge (APC). Authors will not be required to make a final decision about access to their article until it has been accepted. Find out more about Transformative Journals

<B>Authors may need to take specific actions to achieve compliance with funder and institutional open access mandates. For submissions from January 2021, if your research is supported by a funder that requires immediate open access (e.g. according to Plan S principles) then you should select the gold OA route, and we will direct you to the compliant route where possible. For authors selecting the subscription publication route our standard licensing terms will need to be accepted, including our self-archiving policies. Those standard licensing terms will supersede any other terms that the author or any third party may assert apply to any version of the manuscript.

In approximately 10 business days you will receive an email with a link to choose the appropriate publishing options for your paper and our Author Services team will be in touch regarding any additional information that may be required.

You will not receive your proofs until the publishing agreement has been received through our system.

If you have any questions about our publishing options, costs, Open Access requirements, or our legal forms, please contact ASJournals@springernature.com

Sincerely,

Carolina

Carolina Perdigoto, PhD
Chief Editor
Nature Structural & Molecular Biology
orcid.org/0000-0002-5783-7106
